# Regulatory Challenges and Opportunities for Cell‐Derived Extracellular Vesicles in Pharmaceutical Development: A European and Global Perspective

**DOI:** 10.1002/jev2.70332

**Published:** 2026-06-29

**Authors:** Tania Limongi, Paola Milla, Barbara Stella, Silvia Arpicco

**Affiliations:** ^1^ Department of Drug Science and Technology University of Turin, Via P. Giuria 9 Turin Italy

## Abstract

Cell‐derived extracellular vesicles (EVs) attract growing interest as biologically active, acellular platforms for therapeutic and diagnostic use in regenerative medicine, immunomodulation, and drug delivery. While EV‐based products advance into clinical development worldwide, their acellular nature maps non‐uniformly onto pre‐existing categories. In the European Union, the 2025 European Medicines Agency/Committee for Advanced Therapies (EMA/CAT) guideline clarifies that “not substantially modified extracellular vesicles” fall outside the current advanced therapy medicinal products (ATMPs) definition, requiring case‐by‐case development within other medicinal‐product frameworks. Conversely, the United States Food and Drug Administration (FDA) regulates exosome/EV products for disease treatment as drugs and biological products subject to premarket requirements, while other regions apply existing drug and regenerative‐medicine–related instruments using jurisdiction‐specific classification criteria. This heterogeneity creates challenges for regulatory positioning, quality assessment, comparability, and cross‐regional clinical development. We critically examine the evolving landscape of EV‐based therapeutics, focusing on regulatory, technical, safety, and ethical considerations from a global perspective. We argue EV‐based products should be developed within existing medicinal product frameworks (e.g., biologics, ATMP‐related instruments), provided regulatory tools are applied consistently, and operational grey zones (e.g., classification criteria, critical quality attributes (CQAs), potency strategies, and comparability) are identified for clarification. Without proposing normative frameworks, this work provides a state‐of‐the‐art synthesis intended to support ongoing regulatory dialogue across regions. The discussion is relevant for international agencies such as the EMA, FDA, the Pharmaceuticals and Medical Devices Agency (PMDA) of Japan, the World Health Organization (WHO), and the International Council for Harmonisation of Technical Requirements for Pharmaceuticals for Human Use (ICH), as well as for scientific societies such as the International Society for Extracellular Vesicles (ISEV) promoting methodological convergence. By aligning scientific insight with regulatory awareness, this review supports advancing EV‐based therapies toward safe, reproducible, and internationally credible clinical use.

## Graphical abstract

EV‐based therapeutics are advancing worldwide, but regulatory classification and development expectations remain inconsistent across jurisdictions. This review maps challenges in quality, potency, comparability, safety, clinical design, and ethical/legal oversight, and argues for consistent use of existing medicinal‐product frameworks. We emphasize the International Council for Harmonisation of Technical Requirements for Pharmaceuticals for Human Use (ICH) guidelines as a shared baseline and encourage cross‐agency dialogue to develop aligned points‐to‐consider through harmonization channels, including ICH and World Health Organization (WHO) mechanisms, enabling translation.

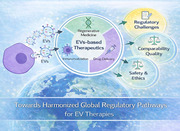

**Abbreviations**
AE: Adverse Event
APPI: Act on the Protection of Personal Information
ARM: Alliance for Regenerative Medicine
ATMP: Advanced Therapy Medicinal Product
BD: Biodistribution
CAT: Committee for Advanced Therapies
CD63: Cluster of Differentiation 63
CD81: Cluster of Differentiation 81
CD9: Cluster of Differentiation 9
CDSCO: Central Drugs Standard Control Organization
CMC: Chemistry, Manufacturing, and Controls
CQA: Critical Quality Attribute
DCGI: Drugs Controller General of India
DNA: Deoxyribonucleic Acid
EC: European Commission
ELISA: Enzyme‐Linked Immunosorbent Assay
EMA: European Medicines Agency
EU: European Union
EV: Extracellular Vesicle
FBS: Fetal Bovine Serum
FDA: Food and Drug Administration
GCP: Good Clinical Practice
GDP: Good Distribution Practice
GDPR: General Data Protection Regulation
GLP: Good Laboratory Practice
GM130: Golgi Matrix Protein of 130 kDa
GMP: Good Manufacturing Practice
HIPAA: Health Insurance Portability and Accountability Act
HLA: Human Leukocyte Antigen
ICH:  International Council for Harmonisation of Technical Requirements for Pharmaceuticals for Human Use
II: Phase II (clinical trial)
III: Phase III (clinical trial)
IND: Investigational New Drug
ISCT: International Society for Cell & Gene Therapy
ISEV: International Society for Extracellular Vesicles
ISO: International Organization for Standardization
IV: Phase IV (clinical trial)
JSEV: Japanese Society for Extracellular Vesicles
JSRM: Japanese Society for Regenerative Medicine
MFDS: Ministry of Food and Drug Safety
MHRA: Medicines and Healthcare products Regulatory Agency
MISEV: Minimal Information for Studies of Extracellular Vesicles
MISEV2014: MISEV Guidelines 2014
MISEV2018: MISEV Guidelines 2018
MISEV2023: MISEV Guidelines 2023
MoA: Mechanism of Action
MSC: Mesenchymal Stromal Cell
MTT: Methylthiazolyl Diphenyl‐Tetrazolium Bromide
NDCTR: New Drugs and Clinical Trials Rules
NK: Natural Killer
NTA: Nanoparticle Tracking Analysis
PK: Pharmacokinetics
PMDA: Pharmaceuticals and Medical Devices Agency
PTEN: Phosphatase and Tensin Homolog
RNA: Ribonucleic Acid
SEC: Size Exclusion Chromatography
SoHO: Substances of Human Origin
TEM: Transmission Electron Microscopy
TFDA: Taiwan Food and Drug Administration
TFF: Tangential Flow Filtration
TGA: Therapeutic Goods Administration
TSG101: Tumor Susceptibility Gene 101 Protein
UF: Ultrafiltration
UNESCO: United Nations Educational, Scientific and Cultural Organization
WHO: World Health Organization
WST‐1: Water Soluble Tetrazolium Salt‐1

## 1 Introduction

Ensuring the quality of extracellular vesicle (EV)‐based therapeutics is essential to guarantee their safety, efficacy, and reproducibility. However, the intrinsic complexity and heterogeneity of EVs ‐combined with the absence of universally accepted analytical and functional standards‐ still pose substantial challenges for regulatory compliance. These challenges do not stem from a lack of regulatory instruments per se, but rather from the difficulty of consistently interpreting and applying existing regulatory frameworks, originally developed for biologics, advanced therapies, and other complex medicinal products, to acellular vesicle‐based systems. Within the European regulatory framework, the 2025 European Medicines Agency/Committee for Advanced Therapies (EMA/CAT) guideline on investigational advanced therapy medicinal products (ATMPs) provides general references for quality standards applicable to cell‐derived products (Food and Drug Administration 1987). However, as clarified in the “Scope” section of the same guideline, EVs, particularly those not substantially modified, are explicitly excluded from the current ATMP classification. This exclusion does not imply that EVs fall outside pharmaceutical regulation, but rather that their development must be framed within existing medicinal product categories (e.g., biologics), using a case‐by‐case, risk‐based interpretation of available guidance.

Beyond the European Union, EV‐based therapeutics are similarly regulated within established pharmaceutical frameworks rather than through EV‐specific legislation. In North America, the United States provides a clear example: the Food and Drug Administration (FDA) regulates exosome‐ and EV–based products intended for the treatment of diseases or conditions in humans as drugs and biological products under the Public Health Service Act and the Federal Food, Drug, and Cosmetic Act. This position has been explicitly reiterated in the FDA's Public Safety Notification on Exosome Products, which clarifies that such products are subject to pre‐market review and approval requirements when therapeutic claims are made (Food and Drug Administration 2019). Consistent with comparative summaries, EV‐based therapeutics in the United States are advanced through the investigational new drug (IND) pathway and reviewed within the general biological drug framework through FDA structures for biologics; developers are expected to provide detailed manufacturing and characterization information and to justify product heterogeneity as an inherent biological feature that can remain compatible with clinical development when appropriately controlled and supported by pharmacological and toxicological evidence (Li et al. 2025). Canada further illustrates how EV‐containing products can be governed through established legal definitions and classification principles. A particularly illustrative example is provided by the Notice issued by Health Canada on the classification of topical products containing human‐derived exosomes, human EVs, and/or human cell‐conditioned media (14 August 2025). This document clarifies that classification is the first step of the regulatory process and is determined under the Food and Drugs Act based on a product's function, purpose, and representation for use, rather than on the mere presence of EVs. Accordingly, topical products containing human‐derived EVs may be regulated either as cosmetic products or as drug products, depending on established criteria such as therapeutic claims, level of biological action, percutaneous absorption, route of administration, overall composition, and related considerations. Products making therapeutic, pharmacological, or biological claims, or intended to restore, correct, or modify organic functions, are classified as drugs and are subject to the Food and Drug Regulations, including pre‐market authorization requirements (e.g., No Objection Letter for clinical trials, Notice of Compliance and Drug Identification Number for marketing). By contrast, products with purely cosmetic claims and no systemic or significant biological action may fall under the Cosmetic Regulations (European Union 2007). In East Asia, EV‐based products are likewise addressed through existing regulatory instruments, but jurisdiction‐specific classification criteria and implementation pathways contribute to practical heterogeneity. In Japan, EV‐based products that do not contain living cellular components are addressed within existing drug and regenerative medicine–related regulatory frameworks under the oversight of the Pharmaceuticals and Medical Devices Agency (PMDA). Comparative analyses indicate that EVs lacking viable cells are generally treated as drugs rather than as “regenerative medical products” and that non‐commercial clinical research conducted independently by medical practitioners may fall outside the scope of the Act on the Safety of Regenerative Medicine when EVs are not classified as specified processed cells (Li et al. 2025). In China, regulatory practice has evolved through multiple pathways, and certain EV‐based therapies have been classified within advanced‐therapy or cell‐related regulatory categories, including advanced therapy medicinal product (ATMP)‐equivalent categories, depending on product characteristics, intended use, and clinical context. Under earlier product registration approaches reported particularly in China, some stem cell‐derived EV applications were handled as “medical technologies” with hospital‐centered accountability models, which facilitated clinical use but posed challenges for broader industrialization and standardized oversight (Li et al. 2025). Additional jurisdiction‐specific developments further illustrate this heterogeneity: South Korea has introduced or issued EV‐relevant regulatory initiatives under the Ministry of Food and Drug Safety, while Taiwan regulates regenerative‐medicine products through a dedicated legislative framework under the Taiwan FDA, with structured approaches to product categorization, clinical oversight, and implementation pathways. Beyond North America and East Asia, other regulatory systems similarly govern EV‐based interventions through established medicinal‐product and clinical‐trial instruments. In India, EV‐based therapies are regulated within existing frameworks for biological medicinal products and clinical research under the oversight of the Central Drugs Standard Control Organization and the Drugs Controller General of India, with first‐in‐human studies requiring prior authorization and ethics review under the New Drugs and Clinical Trials Rules (NDCTR, 2019); in this context, there is a strong focus on manufacturing quality, safety, immunogenicity, and pharmacokinetics before trial initiation. In Switzerland, the Swiss Agency for Therapeutic Products (Swissmedic) applies a stringent, risk‐based evaluation aligned with advanced‐therapy oversight for biological and cell‐derived products, including those derived from human tissues and cells, thereby supporting regulatory convergence with European standards while maintaining a national authorization pathway. In the United Kingdom, following Brexit, the Medicines and Healthcare products Regulatory Agency retains regulatory approaches closely aligned with EMA and International Council for Harmonisation of Technical Requirements for Pharmaceuticals for Human Use (ICH) guidance; EV‐based products are assessed on a case‐by‐case basis within existing frameworks for biological medicinal products and advanced therapies, with particular emphasis on manufacturing quality, safety, and clinical justification. Similarly, the Australian Therapeutic Goods Administration (TGA) regulates EV‐based products within established medicines and biologicals frameworks, applying internationally harmonized standards for quality, non‐clinical evaluation, and clinical development (Verma and Arora 2025).

Collectively, these regulatory practices indicate that EV‐based therapeutics are already being actively governed across multiple jurisdictions through existing pharmaceutical legislation, albeit with differences in classification criteria and implementation pathways. Comparative analyses further suggest that EVs are generally not treated as a separate review class, but are instead assessed within existing biological drug frameworks or, depending on therapeutic context and perceived clinical value, through established accelerated, fast‐track, conditional, or otherwise facilitated development pathways. At the same time, EV‐containing products may enter non‐drug regulatory pathways, such as cosmetics, medical technologies, or medical devices, depending on jurisdiction and product claims, contributing to a heterogeneous and sometimes fragmented regulatory landscape. Notably, EV‐containing products are widely marketed in cosmetic settings internationally, yet most major jurisdictions have issued limited EV‐specific guidance for cosmetics, and the boundary between cosmetic and medicinal claims remains an area of practical regulatory uncertainty. Published comparisons further note that the US FDA has not formally categorized EVs as cosmetics, leaving uncertainty on the appropriate regulatory approach for EV‐based cosmetic products, and that, despite growing marketing, the regulation of EV‐based cosmetics remains largely absent in Japan and South Korea (Li et al. 2025).

Across these jurisdictions, the ICH provides a shared technical foundation for the development and regulatory assessment of medicinal products, including biologics. In particular, ICH quality guidelines addressing viral safety, characterization, comparability, and manufacturing consistency of biological substances (e.g., ICH Q5A–Q5E) are directly relevant to EV‐based therapeutics and are already applied in regulatory practice across Europe, the United States, and Japan (European Union 2024, International Council for Harmonisation 2005).

In this context, the International Society for Extracellular Vesicles (ISEV‐ https://www.isev.org) has emerged as a central actor in fostering scientific consensus and methodological harmonization within the EVs research community. Through the coordinated development of expert guidelines, inter‐society position papers focused recommendations on critical aspects such as EVs isolation, characterization, and functional evaluation, ISEV has significantly contributed to improving the transparency and reproducibility of EVs studies (Pharmaceuticals and Medical Devices Agency 2018; Food and Drug Administration 2020; Schürz et al. 2022; Van Royen et al. 2023; Welsh et al. 2024). The ongoing efforts of its Rigor and Standardization Subcommittee ‐including thematic task forces and special interest groups‐ are particularly relevant for advancing source‐specific best practices.

These community‐driven initiatives, including the Minimal Information for Studies of Extracellular Vesicles (MISEV2023) (Welsh et al. 2024), do not constitute regulatory requirements but provide scientifically robust reference frameworks that can support regulatory dialogue and quality strategy development when aligned with existing regulatory guidance.

In the following sections, we systematically dissect the regulatory, technical, and ethical pillars underpinning the development of EV‐based therapeutics, adopting a genuinely global perspective that considers how existing regulatory instruments are applied across different jurisdictions. The analysis begins with a detailed assessment of essential quality attributes, including identity, purity, potency, and batch‐to‐batch consistency, within the context of their integration into Good Manufacturing Practice (GMP)‐compliant workflows and investigational product release specifications. This is followed by a focus on safety‐related issues—notably immunogenicity, tumorigenicity, off‐target biological effects, and the pharmacological consequences of systemic biodistribution and on the implementation of long‐term pharmacovigilance plans.

We also examine how preclinical and clinical development strategies are being adapted in light of existing regulatory guidance and accumulated clinical experience across regions, including Europe, the United States, Japan, China, and the United Kingdom, addressing challenges related to dosing, potency assays, comparability, and regulatory classification. Ethical and legal considerations‐ such as donor consent traceability, compliance with the General Data Protection Regulation (GDPR), and the proliferation of unauthorized EV‐based interventions outside regulatory oversight‐ are also reviewed.

In the current absence of EVs‐specific regulatory definitions, stakeholders are required to operate within existing categories applicable to biologics and ATMPs, often resorting to scientific advice procedures and regulatory dialogue to resolve classification uncertainties. Importantly, regulatory challenges for EV‐based products in medical and pharmaceutical applications stem less from a lack of legal instruments than from inconsistent and non‐transparent interpretation and implementation of existing frameworks across jurisdictions. Progress therefore depends on regulatory convergence, sustained scientific dialogue, and shared technical standards, rather than on creating new regulatory regimes. In this context, this review article emphasizes the consistent, science‐based application of existing regulatory frameworks to EV‐based products in clinical applications, while transparently identifying recurring interpretative and operational gaps and fostering constructive, globally relevant dialogue among regulators, developers, and the scientific community.

## 2 Quality Requirements of EV‐Based Products

The establishment of appropriate quality requirements is a central element in the development of EV‐ based products, as it underpins safety, efficacy, and reproducibility throughout the product lifecycle. From a regulatory perspective, EV‐based therapeutics are assessed within existing quality frameworks applicable to biological medicinal products, and the primary challenge lies in translating established quality principles to vesicle‐based systems in a scientifically robust and product‐specific manner, rather than in the absence of applicable regulatory standards. Across major regulatory jurisdictions, including Europe, the United States, and Japan, internationally harmonized quality concepts developed under the ICH provide a shared foundation for the assessment of identity, purity, potency, and comparability of complex biological products, and are directly relevant to EV‐based therapeutics.

### 2.1 Identity and Characterization

The characterization of EVs identity must rely on multiparametric strategies, in accordance with the recommendations set by the MISEV2023 guidelines. Positive markers, such as the tetraspanins CD9, CD63, and CD81, along with proteins like TSG101, should be detected to confirm the presence of vesicular structures. At the same time, the absence of negative markers such as calnexin and GM130 must be demonstrated, to exclude contamination by cellular debris (Welsh et al. 2024). In parallel, morphological and size‐based assessments, performed using transmission electron microscopy (TEM), nanoparticle tracking analysis (NTA), and flow cytometry, contribute to defining the vesicular nature of the product. In addition, guidance developed in Japan in cooperation between the Japanese Society for Regenerative Medicine (JSRM‐ https://en.jsrm.jp) and the Japanese Society for Extracellular Vesicles (JSEV‐ https://jsev.jp) explicitly emphasizes that verification should address both (i) the presence of particles with the expected EV‐like size/shape and (ii) EV marker molecules, and notes that some approaches (e.g., nano‐flow cytometry, immunoelectron microscopy, and single‐EV immunodetection) can support the simultaneous assessment of these two elements when feasible (Terai et al. 2025). These complementary methods establish a specific EVs signature, enabling distinction from non‐vesicular contaminants like lipoproteins or protein aggregates. In line with regulatory approaches applied to complex biologics, EV identity should be defined in a fit‐for‐purpose manner, considering the intended use, manufacturing process, and proposed mechanism of action (MoA) of the product. As EV preparations often consist of heterogeneous vesicle populations, identity determination typically requires the integration of orthogonal analytical methods rather than reliance on a single marker or technique. To enhance the robustness of EVs characterization, the use of orthogonal methods is strongly encouraged, combining biochemical, biophysical, and functional analyses. Recent advancements in single‐vesicle technologies, such as fluorescence‐based NTA and nano‐flow cytometry, also offer the possibility to resolve EVs subpopulations that may have particular therapeutic relevance (Sorrells et al. 2024; Basso et al. 2024). From a regulatory‐facing quality perspective, this type of single‐vesicle readout can be positioned as supportive evidence to better link “what is present” (particle populations and marker‐defined fractions) to “what matters” for the specific product, provided that the method is used in a fit‐for‐purpose way and interpreted together with orthogonal biochemical and imaging evidence (rather than as a standalone identity claim). However, the interpretation of data generated by these platforms is highly dependent on instrument settings, acquisition parameters, and data analysis strategies, underscoring the importance of transparent method reporting and careful contextualization when such data are used to support regulatory decision‐making. Building on this point, Terai et al. explicitly frame EV characterization for clinical application as a fit‐for‐purpose exercise in which the performance and limitations of each analytical platform must be understood, documented, and controlled, particularly for single‐particle/single‐vesicle readouts (e.g., fluorescence‐based NTA and nano‐flow cytometry). In this context, the establishment of standardized acquisition and analysis settings and instrument‐specific standard operating procedures becomes critical to reduce inter‐laboratory variability and to avoid over‐interpreting apparent “subpopulations” that may be strongly influenced by thresholds, calibration strategies, and analysis pipelines rather than by true biological differences (Terai et al. 2025). Consistently, the ISEV/ISCT position paper emphasizes that identity and characterization strategies for mesenchymal stromal cell‐derived small extracellular vesicles (MSC‐sEV) therapeutics should be anchored to the intended clinical function and manufacturing control strategy, combining orthogonal methods and transparent reporting to support comparability and product understanding across development phases (Therapeutic Goods Administration 2024). In this respect, recent regulator‐facing guidance developed in Japan emphasizes that single‐vesicle and fluorescence‐enabled platforms should be supported by clearly defined acquisition thresholds, calibration and controls, and, where feasible, pre‐established operating procedures to mitigate instrument‐dependent variability when such datasets are used to substantiate identity or support comparability arguments. The same guidance also highlights that, when identity claims rely on detection of specific markers at the single‐vesicle level, the evidentiary strength depends not only on the marker panel but also on the analytical context (e.g., background subtraction strategies, gating logic, and sensitivity limits), which should be documented in a way that is interpretable for regulatory review (Terai et al. 2025). From a quality and comparability standpoint, single‐particle/single‐vesicle readouts should be interpreted with particular caution when they depend on fluorescent membrane dyes or platform‐specific gating strategies. Multiple studies have shown that commonly used lipophilic dyes can introduce confounding artefacts in uptake and tracking experiments (including dye aggregation, micelle formation, or signal contributions not uniquely attributable to EVs), and that labeling strategies may affect apparent EV size distributions measured by NTA. Accordingly, when fluorescence‐based NTA or nano‐flow cytometry are used to support identity or subpopulation claims, developers should report instrument settings, thresholds, calibration approaches, and analysis pipelines in a transparent and reproducible manner, ideally supported by orthogonal confirmation methods (Therapeutic Goods Administration 2024).

### 2.2 Purity: Reducing Contaminants and Biological Noise

Purity represents a critical hurdle in the development of EV‐based products. Contaminants frequently found in EVs preparations include serum proteins, particularly those derived from fetal bovine serum (FBS), extracellular RNA and DNA, lipoproteins, apoptotic bodies, and residual components from culture media. In addition, regulator‐facing discussions have specifically stressed that, when FBS or other biologically sourced supplements are used, developers should not only qualify source materials for adventitious agents but also assess whether medium additives (including serum‐derived components) are detectable in the final EV preparation and demonstrate their sufficient elimination or reduction by downstream processing (Terai et al. 2025).

Various purification strategies, including ultracentrifugation (UF), size‐exclusion chromatography (SEC), immunoaffinity isolation, and tangential flow filtration (TFF), have been applied to enhance EVs purity, although these methods differ significantly in scalability, reproducibility, and compatibility with GMP production requirements (Welsh et al. 2024; Lau et al. 2022; Costa‐Ferro et al. 2024). Within the framework of ATMPs, the EMA outlines the necessity for consistent removal of impurities with known or potential biological activity, particularly for products intended for systemic or repeated administration (Food and Drug Administration 1987). The use of xeno‐free and chemically defined media is also recommended to minimize the risks of zoonotic transmission and immunogenicity. Recent guidance has further clarified that animal‐derived excipients (and, more broadly, biologically sourced excipients) may introduce viral contamination risks and that bovine EVs present in serum supplements may co‐purify with the product, potentially confounding biological activity; accordingly, the use of reagents free of animal‐derived substances or based on recombinant components is recommended when feasible. Concrete examples of serum‐borne substances that have been highlighted as potentially relevant impurities include albumin, fibrinogen, fibronectin, filamin, apoB, macroglobulin, hemoglobin, and other blood coagulation factors, reinforcing the need for a risk‐based, analytically supported impurity control strategy (Terai et al. 2025).

Although these recommendations were developed for cell‐based therapies, they can be pragmatically and intuitively applied to EVs, which ‐being secreted by living cells and recoverable from cell culture supernatants, tissues, or patient‐derived biological fluids‐ share many of the same safety and manufacturing challenges. From a broader regulatory standpoint, impurity control strategies for EV‐based products are conceptually aligned with quality principles applied to biologics across jurisdictions, including risk‐based assessment of process‐ and product‐related impurities, evaluation of their potential biological activity, and implementation of appropriate control strategies. Such principles, reflected in internationally harmonized guidance for biological products, support a consistent approach to purity assessment without necessitating EV‐specific regulatory requirements. Beyond “classical” process‐related impurities, both Terai et al. (2025) and the ISEV/ISCT position paper highlight that co‐isolated non‐vesicular entities and loosely associated components can materially affect apparent EV activity and therefore confound both purity assessment and downstream interpretation of potency. In practical development terms, this means that purity specifications should be linked to the intended MoA and to clinically relevant safety risks, with control strategies justified by the manufacturing process and the intended route/regimen of administration (Terai et al. 2025; Giebel and Lim 2025). In practice, this reinforces the need for fit‐for‐purpose impurity specifications and control strategies that are aligned with the intended clinical use (including route, dose, and repeat administration), and that are supported by appropriately designed controls to distinguish EV‐driven effects from those driven by process‐related or co‐isolated material.

## 3 Manufacturing Process and GMP Compliance

At present, the implementation of GMP principles in the production of EV‐based therapeutics remains an emerging and dynamic area, shaped by both the biological complexity of EVs and by the need to translate established GMP frameworks for biological medicinal products to vesicle‐based systems, rather than by the absence of applicable regulatory standards. Across major regulatory jurisdictions, including Europe, the United States, and Japan, GMP requirements for biologics constitute the primary reference framework for EV manufacturing, and can be applied when interpreted in a product‐specific and risk‐based manner. Established GMP and quality system principles for biologics (and, where relevant, ATMP‐related manufacturing expectations) therefore provide a robust basis for development when applied consistently and transparently. Efforts to address this gap have come from a growing number of scientific contributions that provide practical guidance on early‐phase manufacturing, quality control, and clinical translation of EV‐based products (Lau et al. 2022; Costa‐Ferro et al. 2024; Thakur and Rai 2024; Takakura et al. 2024; Wang et al. 2024; Wiest and Zubair 2025). While the ICH continues to provide the global foundation for pharmaceutical GMP principles, national regulatory agencies have begun to articulate context‐specific expectations for EV‐based products within existing frameworks. In this regard, important and forward‐looking contributions have emerged from Japan. In particular, the PMDA, together with the JSRM, has taken proactive steps to discuss quality and safety considerations applicable to EV‐based products.

The *Guidance on the clinical application of* EVs by Terai et al. (2025) represents one of the most structured efforts to contextualize GMP principles for EV‐based applications, illustrating how existing regulatory concepts may be operationalized without introducing EV‐specific GMP requirements, and reflecting Japan's leading role in this evolving field (Terai et al. 2025). In this regulatory context, manufacturers are required to define production workflows that are scientifically robust and sufficiently flexible to accommodate evolving regulatory expectations. This includes the early identification of intended clinical use and target population, which informs key decisions related to scale‐up feasibility, product comparability, and positioning within therapeutic strategies. Such considerations are consistent across jurisdictions and are central to regulatory interactions with agencies such as the EMA, FDA, and PMDA. For instance, EV‐based products designed for rare diseases may follow distinct development and regulatory pathways compared to those intended for high‐prevalence conditions.

Equally critical is the development of a manufacturing process capable of delivering consistent, reproducible outcomes across batches. This involves the establishment of master and working cell banks, the standardization of culture conditions and downstream processing, and the application of validated analytical methods to assess parameters such as particle size and concentration, biomolecular content (e.g., proteins and RNAs), and biological activity.

Defining the route of administration and the therapeutic context whether EVs are used as monotherapies, delivery vehicles, or combination products is another essential aspect, as it directly impacts formulation choices, safety evaluations, and clinical protocols. In the case of human‐derived EVs, a rigorous risk‐based approach is needed to manage donor variability, potential immunogenicity, and the presence of adventitious agents, all of which must be addressed through traceable and validated GMP‐compliant procedures. The following sections further explore the practical and regulatory implications of GMP application to EVs, including facility requirements, critical manufacturing steps, scalability challenges, traceability systems, and the risks associated with non‐compliance and unregulated practices.

### 3.1 GMP Applicability and Facility Requirements

Cell‐derived EVs, despite their acellular nature, are regarded as biologically active products and therefore must be manufactured within controlled GMP‐compliant environments. This implies production in cleanrooms compliant with ISO class specifications, using closed, sterile, and disposable systems to minimize contamination risks (Lener et al. 2015; Lamparski et al. 2002). These expectations are aligned with GMP principles applied to biological medicinal products across regulatory regions and do not imply the need for EV‐specific facility standards. Every production step must be documented through electronic batch records with full traceability. Personnel engaged in EVs manufacturing must be qualified and specifically trained in GMP principles. The design of the manufacturing process must ensure that clinical‐grade EVs products administered to patients consistently meet predefined standards for safety, identity, and quality. From a regulatory perspective, this consistency is assessed within existing inspection and oversight mechanisms applied to biologics, including verification of facility suitability, environmental monitoring, and quality system performance. Before the initiation of clinical trials, developers must therefore prepare for facility inspection and certification by the national competent authorities, following procedures that are conceptually comparable across EMA, FDA, and PMDA regulated contexts (Thakur and Rai 2024; Marcianti et al. 2025; Zhou et al. 2025; Lee et al. 2025).

### 3.2 Critical Manufacturing Steps

The EMA outlines several key steps that are crucial for GMP‐compliant EVs production (European Union 2024; Takakura et al. 2024). Comparable manufacturing expectations are also applied by other regulatory authorities when EV‐based products are developed as biological medicinal products, reflecting a shared risk‐based approach across jurisdictions. It starts with the qualification and banking of donor cells, which must undergo stringent screening for infectious agents and genetic abnormalities to guarantee starting material safety. Such controls are consistent with internationally harmonized principles for biological starting materials, including those reflected in ICH guidance, and are applied irrespective of whether EVs fall within ATMP‐related frameworks or other biologics pathways. Culturing these cells must be performed using xeno‐free and chemically defined media to reduce both immunogenicity and the risk of zoonotic transmission. While these measures are well established in cell‐based manufacturing, their relevance extends to EV‐based products given their derivation from living cells and their potential for systemic or repeated administration. Once cells are cultured, EVs must be isolated and purified using reproducible and scalable techniques. Suitable methods include UF, TFF, SEC, and affinity‐based isolation. The choice among these technologies is typically guided by considerations of scalability, robustness, and compatibility with downstream quality requirements, rather than by EV‐specific regulatory prescriptions. During production, in‐process controls should be defined in a fit‐for‐purpose and process‐specific manner to continuously monitor process performance and product consistency. At a minimum, such controls may include verification of source‐material and donor‐cell qualification where applicable, monitoring of critical culture parameters (e.g., cell viability, morphology, density, passage or culture age, and relevant environmental conditions), control of harvest timing and collection conditions, assessment of purification‐step performance, and monitoring of process‐related indicators linked to yield, purity, and stability. Where relevant, additional controls may include microbial or endotoxin‐related monitoring, predefined acceptance criteria for critical manufacturing steps, and documentation of batch‐to‐batch consistency across the full workflow. In line with GMP principles applied to other complex biologics, the specific in‐process control strategy should be justified according to the manufacturing platform, intended clinical use, and critical quality attributes (CQAs) of the EV‐based product. In line with GMP principles for biologics, these controls support early detection of process variability and form the basis for demonstrating batch‐to‐batch consistency. The final phases involve careful storage and formulation of the EVs under stability‐indicating conditions, such as maintaining them at −80°C or employing lyophilization processes with cryoprotectants to preserve vesicle integrity. Stability strategies are selected on a case‐by‐case basis and are evaluated within existing regulatory frameworks for biological products, including assessment of their impact on identity, potency, and safety over time. Every step of the process must be comprehensively documented and validated according to standards applicable to ATMPs and biologicals. More broadly, manufacturing validation and documentation requirements for EV‐based products align with established expectations for biological medicinal products across regions, supporting regulatory review without necessitating EV‐specific manufacturing standards.

### 3.3 Scalability and Technological Bottlenecks

The scalability of EVs production remains a significant technological bottleneck. Most existing isolation methods were initially developed for research purposes and are not readily adaptable to clinical‐grade, large‐scale manufacturing. This limitation represents a common challenge for emerging biological products and reflects the need to translate laboratory‐scale processes into robust, GMP‐compliant manufacturing workflows rather than a lack of applicable regulatory principles. To address these limitations, developers are encouraged to adopt closed‐loop bioreactor systems, microfluidics‐based purification technologies, and automated quality control platforms (Thakur and Rai 2024; Witwer et al. 2021). However, while such approaches are increasingly explored, they are also associated with technical and operational challenges that are directly relevant for regulatory feasibility and industrial translation.

From a manufacturing standpoint, closed or semi‐closed bioreactor‐based expansion can enable higher cell densities and more controlled culture conditions compared with planar systems, but scale‐up remains non‐trivial. Reported practical constraints include process complexity and cost, limited flexibility for certain cell types or culture modes, and the difficulty of maintaining consistent EV output and quality during prolonged culture and scale‐up. In addition, bioreactor operation introduces variables that can influence EV yield and composition (e.g., hydrodynamic stress, mixing/aeration regimes, perfusion rates, nutrient gradients, and harvest schedules), requiring careful process characterization and control to ensure batch consistency. In this respect, a review focused on clinical‐grade MSC‐EV manufacturing highlights that automated and closed bioreactor approaches are increasingly used to support scalable production while reducing contamination risks, but also emphasizes the need for robust in‐process controls and comparability strategies when transitioning between culture formats or scaling parameters (Wiest and Zubair 2025).

A complementary illustration of bioreactor‐enabled scale‐up is provided by an open protocol describing a scalable workflow for the production and isolation of natural killer (NK) cell‐derived EV‐based cancer biotherapeutics, where upstream process structuring and downstream standardization are treated as interconnected scalability drivers (St‐Denis‐Bissonnette et al. 2024). Such workflow‐oriented approaches are useful because they make explicit which steps are most sensitive to scale (e.g., upstream cell expansion format, harvest frequency/volumes, and the choice of downstream concentration/purification operations), thereby supporting a more objective assessment of manufacturability within GMP constraints rather than presenting bioreactor adoption as a universally “ready” solution. Further, recent bioreactor engineering work specifically addresses yield limitations by designing culture conditions that increase EV output while preserving product characteristics. For example, a hydrodynamic bioreactor strategy for stem cell EV production reports substantially increased EV yields relative to conventional culture while highlighting the importance of controlling hydrodynamic conditions to avoid compromising EV‐associated attributes relevant to downstream quality assessment (Lenoir et al. 2025). Together, these examples reinforce that closed‐loop or intensified upstream systems can support industrial‐scale generation of EVs, but their suitability must be evaluated on a case‐by‐case basis within existing GMP frameworks, supported by process characterization, predefined acceptance criteria for CQAs, and early dialogue with regulators.

From a regulatory standpoint, scale‐up or transition between manufacturing platforms, including changes in bioreactor format, culture mode, media composition, harvest strategy, or downstream purification, should trigger a structured comparability exercise rather than a narrow evaluation based solely on particle yield or size distribution. Comparability assessments should be designed to determine whether the post‐change product remains sufficiently aligned with the pre‐change product with respect to identity‐related attributes, CQAs, impurity profile, potency, and stability. Depending on the magnitude and nature of the manufacturing change, developers may also need to reassess biodistribution‐relevant properties or provide additional non‐clinical bridging data when analytical comparability alone is insufficient to exclude clinically meaningful differences. In practical terms, this means that comparability for EV‐based products should be approached as a multidimensional exercise integrating orthogonal analytical methods, functional testing, and, where justified, supplementary non‐clinical evidence.

Another critical aspect concerns the choice of container materials, since EVs are highly sensitive to surface adsorption and aggregation. Using siliconized vials or specialized polymer‐based containers can significantly improve EV stability (Thakur and Rai 2024; Witwer et al. 2021).

Furthermore, lyophilization strategies incorporating stabilizing agents like trehalose or sucrose are recommended to ensure the long‐term preservation of vesicle integrity during storage (Costa‐Ferro et al. 2024; Susa et al. 2023; Jansen et al. 2025). As with other biological medicinal products, container–closure systems and storage strategies for EV‐based products are assessed as integral components of the overall quality and stability profile, and their impact on identity, potency, and safety must be justified through appropriate stability studies rather than predefined EV‐specific requirements.

### 3.4 Traceability and Documentation

Full traceability throughout the entire manufacturing process is a fundamental requirement for human‐derived products, and this is no different for EVs. Developers must be able to trace and document donor identification, while maintaining coded anonymization to ensure privacy, along with detailed records of cell expansion activities, batch numbers of culture media and reagents, and all operational parameters employed during EVs isolation and purification. Additionally, conditions of storage, packaging, and distribution must be fully recorded. From a regulatory standpoint, traceability and documentation requirements for EV‐based products are derived from existing frameworks applicable to biological and human‐derived medicinal products and are consistently applied across major regulatory jurisdictions. Such requirements underpin not only product quality and patient safety, but also enable effective root‐cause analysis in the event of deviations or adverse events (AEs). Such traceability is essential not only for the effective investigation of any AEs but also to meet regulatory obligations under EU Regulation 2024/1938 and Directive 2004/23/EC concerning products derived from human tissues and cells (European Union 2004; 2024). While the Substances of Human Origin (SoHO) Regulation is specific to the European Union, comparable traceability expectations are embedded in regulatory frameworks applied by other authorities, including the US FDA and the PMDA of Japan, reflecting a shared emphasis on documentation, donor oversight, and lifecycle traceability for human‐derived products (Food and Drug Administration 2001; European Medicines Agency 2025).

### 3.5 Risk of Non‐Compliance and Unregulated Practices

A major risk facing the EVs field is the growing availability of unregulated EV‐based products, particularly those manufactured outside GMP oversight. The European Commission's 2025 report on unauthorized ATMPs highlights that several EV‐based interventions currently offered through wellness clinics or online providers do not meet even the minimum standards for quality and safety (International Council for Harmonisation 2005). Although this report focuses on the European context, similar concerns regarding unapproved or improperly manufactured EV‐based interventions have been raised by regulatory authorities in other regions, including the United States and Asia, underscoring the global nature of this issue. These products pose significant risks by introducing contamination, batch variability, and immunological hazards, and they contribute to undermining public trust in the therapeutic use of EVs. From a regulatory perspective, such practices do not reflect gaps in existing legislation, but rather failures to comply with established regulatory and GMP frameworks applicable to biological medicinal products. It is therefore imperative that developers of legitimate EV‐based therapies adhere strictly to GMP guidelines and to applicable regulatory oversight mechanisms to avoid regulatory penalties, reputational damage, and, most importantly, to guarantee patient safety.

In conclusion, the establishment of GMP‐compliant, scalable, and traceable manufacturing processes forms the foundation for the successful clinical development of EV‐based therapeutics. Even in the absence of EV‐specific GMP provisions, consistent application of existing GMP and quality system requirements for biologics and ATMPs, together with risk‐based validation strategies and transparent regulatory interactions, provides a robust and internationally convergent pathway for regulatory acceptance. This approach supports the safe and responsible integration of EV‐based therapies into clinical practice while safeguarding patients and preserving confidence in the field.

## 4 Risk Management and Safety Considerations

Although EVs are acellular structures and are generally perceived as safer alternatives to cell‐based therapies, their inherent bioactivity, systemic biodistribution, and molecular cargo introduce distinct safety risks that must be rigorously evaluated throughout pharmaceutical development (Takakura et al. 2024; Chen, Tang, et al. 2025; Chen, Chen, et al. 2025). These risks are not unique to EVs but are characteristic of complex biological medicinal products, and are therefore addressed within existing regulatory risk‐management frameworks applied across jurisdictions. There is a pressing need to ensure that risk‐based safety assessments are implemented coherently throughout the entire development process of EVs products, from donor selection to clinical monitoring, by applying existing regulatory expectations for complex biologics and advanced therapies in a consistent manner, while explicitly identifying EV‐specific scientific questions that may require additional clarification through scientific advice and regulator–developer dialogue rather than through the introduction of new regulatory categories. Across major regulatory regions, including Europe, the United States, and Japan, safety evaluation of EV‐based products is embedded within established benefit–risk assessment paradigms, pharmacovigilance systems, and post‐authorization monitoring frameworks. This convergence supports a globally consistent approach to risk management while allowing product‐specific considerations to be addressed on a case‐by‐case basis.

### 4.1 Quality‐Linked Safety Risks

Many safety issues associated with EV‐based products stem directly from quality‐related factors. Impurities arising during the production process, such as residual DNA, cellular debris, or xenogeneic proteins, can contribute to immunotoxicity, batch‐to‐batch variability, and off‐target biological effects. Particularly, the use of animal‐derived components like FBS introduces critical concerns regarding immunogenicity and the risk of zoonotic pathogen transmission.

Given the increasing convergence of EVs with advanced therapy approaches, there is a growing need to interpret and apply existing risk‐based quality principles and contamination control expectations used for advanced therapies and other complex biologics to EV‐based products, using scientific advice and product‐specific justification where classification boundaries remain uncertain. This approach is consistent with regulatory practices adopted by agencies such as the EMA, FDA, and PMDA, which rely on risk‐based evaluation of product quality and safety rather than on rigid product categorization. The ongoing revision of GMP guidelines for ATMPs, which aims to align manufacturing standards with risk‐based quality systems and contamination control strategies (European Medicines Agency 2025), underscores the importance of extending such principles to EVs. These include the implementation of validated purification methods, robust in‐process controls, and clearly defined release criteria to ensure consistent product safety.

To that end, xeno‐free or chemically defined media should be prioritized in EVs manufacturing for clinical use (Witwer et al. 2021). Furthermore, the definition and monitoring of CQAs, such as particle size, molecular content, and surface marker expression, should be integrated into process analytical control strategies throughout the production workflow, in line with internationally harmonized quality concepts for biological medicinal products, thereby aligning EV‐based products with the same quality and safety expectations required for other complex biological therapies.

### 4.2 Immunogenicity, off‐Target Effects and Systemic Biodistribution

The potential for allogeneic or xenogeneic EVs to provoke immune responses arises primarily due to the presence of membrane‐bound human leukocyte antigens (HLAs), cytokines, or foreign proteins on their surfaces. This immunogenic risk is especially pertinent when EVs are derived from immune cells or tumor cells, as these sources may carry immunostimulatory or immunosuppressive molecules that modulate host immune responses (Lener et al. 2015; Xia et al. 2024). In this context, immunogenicity should be considered a spectrum of potential responses rather than a categorical attribute, and its assessment is typically informed by the intended clinical use, dosing regimen, and route of administration.

To mitigate these risks, developers are advised to utilize xeno‐free, chemically defined culture media to eliminate the contribution of animal‐derived immunogenic proteins. Comprehensive donor screening protocols should be implemented to exclude individuals with immunological risk factors. Such mitigation strategies are consistent with risk‐based approaches applied to other biological medicinal products and are evaluated within existing regulatory frameworks rather than through EV‐specific safety requirements. Additionally, strategies to remove immunogenic surface proteins or engineer immunologically inert EVs can further reduce the likelihood of adverse immune responses. While autologous EVs tend to present a lower risk of immunogenicity (Wu et al. 2025), they are not exempt from safety concerns, as improperly purified or stored autologous EVs may still induce immune activation. Particular attention is warranted in the context of repeated or chronic administration, where cumulative immune responses may emerge even in the absence of acute immunogenicity. Such considerations are routinely addressed for biologics through non‐clinical and clinical immunogenicity assessments and are equally applicable to EV‐based products.

In repeat‐dose or chronic administration settings, the immunological behavior of EV‐based products warrants more systematic consideration, particularly for allogeneic preparations. Although early‐stage studies and several preclinical models have generally suggested favorable short‐term tolerability, unintended immunological recognition remains insufficiently characterized, especially beyond acute exposure. Emerging evidence indicates that allogeneic and xenogeneic EVs may undergo enhanced clearance by both innate and adaptive immune systems after repeated administration. Potential mechanisms include recognition of donor‐associated antigens presented on EV surfaces, particularly major histocompatibility complex (MHC)‐related determinants, antibody development against EV membrane‐associated molecules, complement‐mediated interactions, and immune activation triggered by co‐isolated contaminants or process‐related impurities (Takakura et al. 2024; Xia et al. 2024).

In addition, EV immunogenicity may be modulated by factors such as source and differentiation state of the producer cells, surface composition, internal cargo, production and storage conditions, dosage, infusion rate, and biomolecular corona formation (Xia et al. 2024).

From a regulatory and translational perspective, cumulative immunogenicity in repeat‐dose settings should therefore be approached through a fit‐for‐purpose preclinical strategy, while recognizing the limits of conventional animal models for predicting human immune responses to EVs. Relevant assessments may include repeat‐dose study designs with longitudinal monitoring of immune markers, evaluation of complement activation, cytokine release, immune‐cell phenotyping, phagocytic responses, and, where feasible, assessment of anti‐product antibody formation and changes in circulation time or biodistribution after repeated administration (Xia et al. 2024; Yoo et al. 2022).

Available evidence suggests that repeated dosing may reduce EV circulation time, potentially reflecting accelerated blood clearance phenomena associated with immunological recognition. These considerations support the inclusion of immune monitoring endpoints early in development, particularly when repeated administration is envisioned.

Risk‐mitigation strategies should be aligned with the nature of the EV product and its intended clinical use. These may include careful source selection, minimization of immunostimulatory impurities through controlled manufacturing and purification, avoidance of animal‐derived raw materials where possible, use of xeno‐free or chemically defined media, and consideration of engineering strategies aimed at reducing unwanted immune recognition. In selected allogeneic settings, especially when repeated administration is planned, the potential relevance of MHC compatibility may also merit case‐specific consideration. At the clinical level, cautious dose escalation, attention to infusion conditions, and longitudinal monitoring for immune‐mediated changes in exposure, tolerability, or loss of effect may be warranted. Overall, for EV products intended for chronic indications, the absence of acute immunotoxicity should not be interpreted as excluding cumulative immune risk, and repeat‐dose immunogenicity should be incorporated into the broader benefit–risk evaluation from early development onward (Takakura et al. 2024; Xia et al. 2024).

Due to their small size and lipophilic membranes, EVs are capable of reaching distant organs and influencing off‐target tissues. The biodistribution patterns of EVs are highly dependent on variables such as the cell source, size, membrane composition, and the route of administration. It is well established that following systemic delivery, EVs tend to accumulate predominantly in the liver, spleen, and lungs (Busatto et al. 2021; Su et al. 2025; Gupta et al. 2023; Zhao et al. 2024). These distribution patterns have important implications for both therapeutic efficacy and off‐target safety and must therefore be considered within the overall benefit–risk assessment.

To address and reduce off‐target risks, it is crucial that pharmacokinetic and biodistribution studies are performed early during the development program. These studies should employ validated labeling strategies, including fluorescent or radiolabeling methods, to accurately track EV fate *in vivo*. The selection and validation of labeling approaches are critical, as certain labels may alter EV behavior or dissociate from vesicles, potentially confounding biodistribution data. In addition, developers are encouraged to consider surface modifications that enhance tissue‐specific targeting, such as engineering EVs with targeting peptides or antibodies. While such modifications may improve therapeutic precision, they must also be carefully evaluated for any new safety liabilities that could arise, including altered biodistribution, immunogenicity, or clearance profiles, within existing non‐clinical safety assessment frameworks applied to biologics.

### 4.3 Tumorigenicity and Genetic Material Transfer

Another critical safety consideration is the potential for EVs to contribute to tumorigenesis through the transfer of oncogenic material. EVs, particularly those derived from tumor cells or genetically modified sources, are known to carry oncogenic proteins, microRNAs, and DNA fragments capable of promoting cellular transformation, angiogenesis, or immune evasion. Even EVs originating from non‐transformed donor cells may pose risks if those cells are genetically unstable or have been immortalized (Gao et al. 2022; Bao et al. 2022; Qian et al. 2025; Semeradtova et al. 2025). The relevance of these risks is strongly influenced by the source material, the manufacturing process, and the intended clinical use of the EV‐based product.

Preventive measures include rigorous qualification of donor cells to exclude malignant or pre‐malignant lines. Developers should also implement cargo screening using omics‐based approaches such as RNA sequencing and proteomic analysis to detect the presence of oncogenic factors. Furthermore, dedicated testing must be conducted to assess genotoxicity, transformation potential, and possible epigenetic alterations. Such evaluations are typically integrated into non‐clinical safety assessment programs for biological medicinal products and are applied to EV‐based products using a risk‐based approach, particularly when genetic modification or extensive cell manipulation is involved. These evaluations are particularly important for products intended for repeated or chronic administration, where cumulative risk considerations may become relevant and should be addressed within an appropriate long‐term safety monitoring framework.

The regulatory experience accumulated in the context of cell‐based therapies, which already require comprehensive testing for genomic stability, tumorigenicity, and product consistency, offers a solid foundation for addressing similar concerns in the development of EV‐based products. Accordingly, existing regulatory expectations for advanced therapies and other complex biologics provide a relevant and transferable framework for assessing tumorigenicity and genetic material transfer associated with EV‐based therapeutics.

### 4.4 Long‐Term Monitoring and Pharmacovigilance

Given the novelty and biological complexity of EV‐based therapeutics, long‐term monitoring and enhanced pharmacovigilance measures are indispensable (Lorite et al. 2024; Fusco et al. 2024). As for other complex biological medicinal products, potential delayed or cumulative adverse effects may not be fully captured during early‐phase clinical development, underscoring the importance of lifecycle‐based safety monitoring strategies. In the field of ATMPs, current EMA guidance recommends the integration of risk‐adapted pharmacovigilance plans within development programs to detect, monitor, and manage potential AEs over time. These approaches typically include longitudinal patient registries, biomarker‐based surveillance protocols, systematic AE reporting, and early signal detection systems (European Medicines Agency 2023). Comparable pharmacovigilance principles are embedded in international regulatory practice. The ICH, through guidelines such as ICH E2E on Pharmacovigilance Planning, establishes a global framework for lifecycle‐based safety monitoring applicable to biological medicinal products (International Council for Harmonisation 2005). Similarly, regulatory authorities including the US FDA and the PMDA of Japan require structured post‐marketing safety surveillance, benefit–risk reassessment, and risk management planning for biologics and advanced therapies (Food and Drug Administration 2001, 2018; Lamparski et al. 2002). The Australian TGA likewise adopts internationally harmonized pharmacovigilance standards, aligned with EMA and ICH principles, for biological and gene‐ and cell‐based products (Food and Drug Administration 2024).

While EV‐based therapeutics are currently regulated within existing product categories (e.g., biologics or advanced therapies, depending on their characteristics), and are therefore already subject to the corresponding pharmacovigilance obligations, ATMP‐oriented tools such as enhanced follow‐up, registries, and long‐term safety studies provide a relevant reference model for EV‐based products with comparable risk profiles.

The application of such tools may not only enhance patient safety but also generate critical real‐world evidence to support clinical optimization and regulatory decision‐making over time. Ultimately, the safe clinical implementation of EV‐based therapeutics will likely require a comprehensive and proactive risk management approach. This should include quality‐driven manufacturing processes, a mechanistic understanding of biodistribution and immune interactions, rigorous preclinical evaluation of tumorigenicity and genetic safety, and robust systems for clinical monitoring and post‐administration follow‐up. This lifecycle‐oriented approach is fully consistent with established regulatory practice for complex biologics and advanced therapies and supports the progressive refinement of safety strategies as clinical experience accumulates. Alignment with existing scientific and regulatory standards, such as those outlined in MISEV2023 and those applied to other complex biological medicinal products, may help guide responsible development and clinical translation while informing future evidence‐based regulatory clarification where justified.

## 5 Preclinical and Clinical Development Pathways

The transition of cell‐derived EVs from laboratory research to clinical application necessitates a carefully structured development strategy that integrates rigorous preclinical evaluation with a scientifically sound clinical trial design. As for other complex biological medicinal products, this transition is governed by globally harmonized principles for non‐clinical and clinical development, including those established within the framework of the ICH, and implemented through region‐specific regulatory pathways. Although EVs are not yet explicitly addressed by EV‐specific regulatory guidance, they are currently developed and assessed within existing regulatory categories applicable to biologics or advanced therapies, depending on product characteristics and intended use. Existing guidance for ATMPs and other complex biologics therefore offers a practical foundation for shaping development plans in compliance with current regulatory expectations when applied consistently and justified on a case‐by‐case basis (European Medicines Agency 2023). Importantly, while the overarching scientific and regulatory principles governing preclinical and clinical development are broadly shared across regions, their practical implementation differs among regulatory authorities, including the EMA, the US FDA, the PMDA of Japan, and regulatory agencies in other jurisdictions such as China and Australia. These differences may influence study design, data requirements, and the sequencing of non‐clinical and clinical development steps, underscoring the importance of early regulatory dialogue and region‐aware development strategies. Across guidance and position papers focused on clinical translation, a recurring theme is that EV heterogeneity is not merely a descriptive feature but a development driver that must be actively managed through a clear product concept, a mechanism‐informed quality strategy, and a justified set of CQA linked to identity and potency. This becomes particularly important for repeated or chronic administration scenarios, where immune recognition and cumulative exposure may amplify risks and where a robust MoA rationale is needed to support dose selection, monitoring plans, and benefit–risk evaluation (Terai et al. 2025; Giebel and Lim 2025).

A side‐by‐side comparison of the current regulatory positioning of EV‐based therapeutics across the EMA, the FDA, and the PMDA is useful not only to distinguish areas of genuine convergence from areas in which only broad scientific expectations are shared, but also to make recurring operational grey zones more explicit. Importantly, the EMA column should be interpreted with caution, because the most relevant 2025 EMA/CAT document is a guideline for investigational ATMPs and explicitly states that not substantially modified EVs fall outside the current definition. Accordingly, the comparison below is not intended to imply regulatory symmetry among jurisdictions, but rather to clarify how each authority currently frames product classification, development expectations, quality characterization, potency, and comparability, and how these differences generate practical uncertainties that may affect global development strategies (Table 1).

**TABLE 1:** Comparative analysis of regulatory pathways, classification criteria, and development expectations for EV‐based therapeutics across EMA, FDA, and PMDA.

	EMA (2025)	FDA (2019, 2024)	PMDA (2023), Takakura et al. (2024)	Practical implication for developers
Regulatory framing / classification	Under the 2025 EMA/CAT guideline for investigational ATMPs, not substantially modified EVs do not fulfil the current definition of ATMPs; therefore, the ATMP‐specific risk‐based approach does not automatically apply. The guideline explicitly refers classification questions to the EMA Reflection Paper on ATMP classification.	FDA states that there are currently no FDA‐approved exosome products, and that exosomes used to treat diseases or conditions in humans are regulated as drugs and biological products under the Public Health Service Act and the Federal Food, Drug, and Cosmetic Act, with premarket review and approval requirements.	PMDA has created a dedicated Subcommittee on Therapeutic Products Based on EVs Including Exosomes and published an official EV report. In that framework, PMDA discusses natural EVs and engineered EVs as therapeutic products, rather than as a standalone codified EV legal class.	Product framing is not symmetrical across regions. Developers should not assume that “EVs” map into one universal legal category; classification and development strategy remain jurisdiction‐sensitive.
Regulatory pathway / agency interaction	The EMA document is not an EV‐specific guideline, but it indicates that when development requirements are uncertain, sponsors should consider relevant quality, non‐clinical, and clinical requirements early and seek advice at national or European level, especially if early‐phase trials may later support marketing authorization.	For therapeutic biologics, FDA uses the Biologics License Application (BLA) framework. Biological products may be studied under an IND and, if development is successful, are submitted as a BLA for licensure.	PMDA's official EV initiative explicitly covers evaluation strategies for safety, quality characterization of active ingredients, manufacturing consistency, non‐clinical studies, and clinical trials, which strongly supports an early consultation‐oriented development approach.	A global EV program should be built around early agency dialogue, rather than waiting until late‐stage Chemistry, Manufacturing, and Controls (CMC) or clinical development to resolve regulatory framing.
Characterization / CQAs	Although written for investigational ATMPs, the EMA guideline remains informative by analogy: characterization should address structure, physicochemical properties, biological activity, immunochemical properties, purity, and impurities, and these data should support identification of quality parameters relevant to efficacy and safety.	FDA states that licensure of a biologic requires showing that the product, manufacturing process, and manufacturing facilities meet requirements ensuring safety, purity, and potency. FDA also stresses that biologics are heavily dependent on manufacturing control because of their complexity.	PMDA states that EV characterization should include the manufacturing cells and the EV product itself, including the quality characteristics whose variability may affect efficacy and safety, that is CQAs.	A region‐ready EV dossier should define a fit‐for‐purpose critical quality attribute (CQA) package rather than rely on descriptive marker panels alone. The justification should connect characterization to intended use, safety, and biological performance.
Potency expectations	In the EMA guideline, potency is treated as a quantitative measure of biological activity linked to the relevant biological properties and the claimed MoA`. For EV products, if EMA principles are used by analogy, potency should therefore be handled as a functional, product‐relevant attribute, not merely a descriptive parameter.	FDA explicitly states that a potency assay is required due to the complexity and heterogeneity of biologics, and defines potency as the specific ability or capacity of the product, as indicated by appropriate laboratory tests, to yield a given result.	PMDA is particularly explicit that potency assays should reflect the clinical mode of action. When the mode of action is not fully elucidated, the report recommends a multifaceted approach, using multiple evaluation systems to clarify biological activity.	Potency should be established early and linked to the proposed MoA and quality strategy. It should not be left to the end of development as a release formality.
Comparability after manufacturing changes	If EMA principles are used as a reference point, comparability should follow a stepwise approach comparing processes, active‐substance quality attributes, and relevant intermediates using suitable analytical methods, including orthogonal methods. EMA also states that biological characterization and potency assays are among the most important parameters for comparability, and that if patient risk cannot be excluded, quality comparability alone may be insufficient and additional non‐clinical data may be needed.	FDA states that manufacturing changes may alter biologics in ways not always captured by standard characterization methods, and that changes in process, equipment, or facilities may require additional studies. FDA also states that comparability may be demonstrated through analytical and functional testing when the change does not affect safety, identity, purity, or potency.	PMDA explicitly identifies cell‐bank renewal as a comparability issue, stating that when a cell bank is renewed, assurance of comparability of EV quality before and after renewal becomes an issue. PMDA also emphasizes consistency across the manufacturing culture period.	Scale‐up, media changes, purification changes, cell‐bank renewal, or bioreactor transitions should trigger a multidimensional comparability plan, not just a yield or particle‐count comparison.

As evident from Table 1, the principal area of convergence across jurisdictions lies less in legal categorization than in the scientific expectations applied to clinically intended EV‐based products. At the same time, the comparison makes clear that some of the most relevant grey zones arise precisely at the interface between shared scientific principles and jurisdiction‐specific regulatory interpretation. These include, for example, how EV‐based products are framed within existing regulatory categories, how the degree of manipulation or engineering alters development expectations, which attributes should be treated as product‐relevant CQAs, how potency should be justified when the MoA is only partially resolved, and how comparability should be demonstrated after manufacturing changes. Across regions, developers are therefore expected not only to justify product characterization in a fit‐for‐purpose manner, but also to manage such ambiguities through early regulatory dialogue, transparent justification of development choices, and structured comparability and potency strategies. In this context, the following sections examine how preclinical evaluation, clinical trial design, and potency assessment for EV‐based therapeutics can be aligned with existing regulatory frameworks, while highlighting recurring scientific and operational challenges that must be addressed to support robust, reproducible, and globally acceptable development pathways. A cross‐cutting theme underlying these development steps is that clinically meaningful control of EV heterogeneity depends on a clearly articulated MoA and, where feasible, a more granular MoA, because these elements directly inform the selection of potency assays, the definition of potency‐related CQAs, and the comparability strategy across manufacturing changes (Therapeutic Goods Administration 2024).

### 5.1 Preclinical Evaluation: Pharmacodynamics, Biodistribution, and Toxicology

Preclinical studies of EV‐based products should aim to characterize their mechanisms of action, biodistribution profiles, and toxicological risks. However, EVs complex and context‐dependent biology poses significant challenges. Their cargo ‐consisting of proteins, RNAs, lipids, and metabolites‐ may vary with cell source, culture conditions, and isolation method (Dixson et al. 2023; Payandeh et al. 2024; Wiersema et al. 2024; Hánělová et al. 2024). In line with internationally harmonized non‐clinical development principles, preclinical programs for EV‐based products are expected to be designed using a risk‐ and indication‐driven approach, consistent with guidance applied to other complex biological medicinal products. This context‐dependency has direct implications for how biological activity is interpreted in non‐clinical programs: establishing robust potency‐related CQAs requires an explicit understanding of both the MoA and the MoA underlying the intended therapeutic effect. While MoA describes the broader biological/physiological processes affected (e.g., immunomodulation, tissue repair), the MoA refers to the molecular interactions and targets mediating those effects. Importantly, multimodal MoAs may reflect a combination of direct and indirect pathways, and the relevance of a given *in vitro* readout may depend on the presence of specific responder cell types or microenvironmental conditions. This reinforces the principle that ‘the process defines the product’, and that manufacturing consistency and potency strategy must be co‐developed rather than treated as independent workstreams. Preclinical assessment includes both *in vitro* and *in vivo* studies and is essential for supporting a rational and evidence‐based transition to first‐in‐human trials. *In vitro* assays are often the first step, enabling mechanistic insights and functional screening across relevant cell types. Key readouts include EVs uptake (typically via fluorescent labeling and flow cytometry or confocal microscopy), effects on cell proliferation and survival (e.g., MTT, WST‐1), migration (wound healing assays), and cytokine release or gene expression changes (*via* ELISA, qPCR, or Western blot). These tests help define the biological activity of EVs in the context of the intended indication (Limongi et al. 2021; Dumontel et al. 2022; Li et al. 2022). From a regulatory perspective, such assays contribute to establishing proof of biological activity and support the justification of starting dose and dosing regimen selection for early clinical studies. Consistent with this logic, published Japan‐focused clinical application guidance explicitly emphasizes that *in vitro* and *in vivo* assessment systems should be used to adequately validate the relationship between EV preparation dosage and its safety and effectiveness (Terai et al. 2025). Pharmacodynamic assays should be tailored to the therapeutic context (e.g., immunomodulation, angiogenesis, regeneration), using relevant *in vitro* models and animal species that exhibit human‐compatible EVs uptake (Driedonks et al. 2022; Pharmaceuticals and Medical Devices Agency 2024). *In vivo* studies are employed to validate the efficacy, targeting, and MoA observed *in vitro*. Rodent models (e.g., mouse models of inflammation, ischemia, or fibrosis) and large animals (e.g., porcine models for cardiac or cartilage repair) are used depending on the therapeutic application. These models also help in optimizing dosing, frequency, and administration routes (Pharmaceuticals and Medical Devices Agency 2014; Nørgård et al. 2022; Guo et al. 2025). From a regulatory perspective, comparative summaries underline that clinical protocols are expected to specify route of administration, dosage, and frequency as part of the overall preclinical and clinical development package supporting EV‐based therapeutics (Li et al. 2025).

Consistent with international guidance for non‐clinical development of biological medicinal products, the selection of animal models should be scientifically justified and aligned with the intended clinical use, rather than driven by EV‐specific considerations alone.

Biodistribution studies are essential for determining target organ accumulation and clearance pathways. Systemic administration of EVs has been shown to result in preferential uptake by liver, spleen, and lung tissue, depending on membrane composition and route of delivery (Busatto et al. 2021; Su et al. 2025). Labeling techniques, such as lipophilic dyes or radiotracers, allow tracking of EVs *in vivo* through live imaging or post‐mortem organ analysis. However, limitations include dye leakage, altered EV properties, and insufficient sensitivity to detect low‐abundance signals. Terai et al. further stress that biodistribution readouts should be interpreted in light of method‐specific artefacts (e.g., label dissociation, altered vesicle behavior after labeling, and matrix/background effects) and that the choice of approach should be justified relative to the clinical context and administration route, especially when biodistribution data are used to support safety margins and dose selection (Terai et al. 2025). In line with this, the ISEV/ISCT position paper frames pharmacokinetics (PK, pharmacokinetics) and biodistribution (BD, biodistribution) as integral to the non‐clinical package for MSC‐sEV therapeutics, particularly when repeated administration is envisioned and when linking exposure to functional activity and safety is required to support clinical translation (Therapeutic Goods Administration 2024). These limitations are not merely technical details but can materially affect the interpretability of biodistribution claims used for risk assessment and clinical dose justification. Systematic evaluations and methodological reviews have highlighted that labeling approaches may modify EV apparent size, generate non‐EV‐associated signal, or bias organ‐level quantification, and that the choice of imaging modality (e.g., optical imaging versus positron emission tomography) entails distinct sensitivity and artefact profiles. Consequently, when biodistribution data are used to support first‐in‐human dose selection or off‐target risk arguments, developers should justify the labeling strategy, include appropriate controls for free label and label transfer, and, where possible, triangulate findings using complementary approaches (Therapeutic Goods Administration 2024).

Regulatory authorities generally expect that the strengths and limitations of biodistribution methodologies be clearly characterized and justified, particularly when such data are used to support safety margins and clinical dose selection. Toxicology studies must assess immunogenicity, tumorigenicity, and potential off‐target effects (Yoo et al. 2022; Gupta et al. 2021). Due to the pleiotropic nature of EVs, a “weight of evidence” approach combining *in vitro* and *in vivo* data is encouraged as for ATMPs. *In vitro* assays may include cell viability, apoptosis, oxidative stress, and genotoxicity following EV exposure, while *in vivo* toxicity studies typically involve repeated dosing in rodents with histopathological examination of major organs. International regulatory guidance generally supports the use of at least two dose levels and, where appropriate, inclusion of a recovery period (Food and Drug Administration 2010; Sorrells et al. 2024; European Medicines Agency 2010). Particular attention should be paid to EVs derived from tumor cells or stem cells, where residual oncogenic or pluripotency‐associated signals could pose risks. Even in MSC‐derived EVs, batch‐to‐batch variability in content (e.g., miRNA profiles) may influence both efficacy and safety.

Standardization of protocols, including consistent EVs characterization and potency assays, is crucial to ensure regulatory compliance. Functional tests that correlate with the intended clinical outcome can also support release criteria and comparability assessments during development. Taken together, these preclinical data form a critical component of regulatory submissions across regions and underpin the scientific justification for advancing EV‐based products into clinical evaluation.

### 5.2 Clinical Trials: Design, Safety, and Regulatory Reporting

The promising commercial potential of EVs technology has attracted significant investment, leading to the establishment of numerous companies dedicated exclusively to EVs development. Following encouraging results from preclinical studies, efforts have shifted toward evaluating their clinical applicability. This transition has largely occurred within existing clinical trial and medicinal product regulatory frameworks, with EV‐based investigational products entering clinical evaluation under different regulatory classifications depending on their source, degree of manipulation, and intended therapeutic use. Current clinical research is primarily focused on two fronts: the use of EVs as early, non‐invasive diagnostic biomarkers ‐particularly in oncology‐ and the exploration of their therapeutic potential, especially in regenerative medicine through the use of native EVs. As of February 2026, ClinicalTrials.gov lists over 100 registered interventional trials involving therapeutic use of EVs, ranging from early‐phase safety evaluations to phase III efficacy studies. These trials target a variety of clinical indications, including respiratory failure, inflammatory bowel diseases, neurodegenerative disorders, osteoarthritis, and cancer. While most studies are currently in phase I or phase I/II, an increasing number have progressed to phase II and III, reflecting a growing regulatory confidence and maturation in the clinical development of EV‐based therapies (Mizenko et al. 2024; Camussi and Lötvall 2023). In the European Union, the ethical review, authorization procedure, safety reporting and transparency obligations for clinical trials with EV‐based investigational medicinal products must be conducted in accordance with the consolidate version (5/12/2022) of Clinical Trials Regulation, Regulation (EU) No 536/2014 (European Union 2014) in conjunction with applicable EMA guidance and national implementing provisions. Comparable governance frameworks apply in other regions through established clinical trial and Good Clinical Practice (GCP) requirements, including the U.S. IND framework (21 CFR Part 312, title 21 was last amended 1/22/2026) (Food and Drug Administration 1987) and Japanese GCP under Ministry of Health and Welfare oversight (Ministry of Health and Welfare 2022).

Ongoing clinical trials exploring the therapeutic use of EVs are reported in Table S1. An analysis of the trials summarized in this table highlights that the majority of interventional studies remain exploratory and early‐phase, consistent with a risk‐adapted clinical development strategy typically applied to novel biological medicinal products.

Furthermore, certain types of EVs, either native or loaded with siRNA against phosphatase and tensin homolog (PTEN), have recently been included in both the European and U.S. registries of orphan medicinal products for the prevention of bronchopulmonary dysplasia or for the treatment of spinal cord injuries (European Medicines Agency 2021; European Union 2024).

Institutional sponsorship of EVs trials reflects a strong academic drive, with universities and hospitals leading numerous studies. Private sector involvement is also considerable, with companies like Rion Inc. and Direct Biologics sponsoring several interventional trials.

The clinical landscape summarized in Table S1 further reveals a pronounced heterogeneity in EV sources, including mesenchymal stromal cells derived from bone marrow, adipose tissue, umbilical cord, placenta, neural stem cells, platelet‐enriched blood products, and autologous blood‐derived vesicles. This diversity has direct regulatory implications, as EVs derived from different cellular sources may exhibit distinct biological activities, safety profiles, and manufacturing risks, thereby requiring product‐specific assessment within existing biologics or advanced therapy frameworks.

Notably, the table also documents substantial variability in routes of administration, ranging from intravenous and intra‐articular injection to intranasal, intracoronary, topical, and locally administered EVs. From a regulatory perspective, such variability directly impacts the design of non‐clinical studies, the justification of starting doses and dose‐escalation schemes, biodistribution assessment, and clinical safety monitoring requirements. This point becomes even more consequential when EV‐based products are designed for repeated administration, as cumulative exposure may alter immunological risk profiles and may require fit‐for‐purpose monitoring strategies and a clearer linkage between dose, exposure, and intended biological function, as emphasized in clinically oriented guidance and the ISEV/ISCT position paper on MSC‐sEV therapeutics (Terai et al. 2025; Giebel and Lim 2025). In many cases, however, the publicly available trial documentation does not clearly articulate the rationale linking EV source, route of administration, and intended therapeutic effect. Although EVs have been used in dozens of registered clinical trials to date, methodological inconsistencies persist, resulting in poor standardization of dosing, potency, and quality control (Couch et al. 2021).

A recent review by Mizenko et al. (2024) highlighted that methodological details are largely lacking in the documentation of EVs‐related clinical trials reported on ClinicalTrials.gov, thus compromising reproducibility and regulatory confidence (Mizenko et al. 2024). For example, only a minority of trials explicitly report EV isolation methods, characterization strategies, or cargo profiling, despite the well‐established influence of these parameters on EV biological behavior.

Geographically, the trials reported in Table S1 span Europe, North America, East Asia, the Middle East, and Australia, demonstrating that EV‐based therapeutics are already being developed globally within region‐specific regulatory systems rather than under a unified EV‐specific framework. This global dispersion illustrates both the feasibility of advancing EV‐based products within existing national regulations and the challenges associated with cross‐trial comparability and regulatory convergence.

Overall, the clinical trial landscape summarized in Table S1 indicates that the principal barriers to regulatory confidence are not the absence of applicable regulatory pathways, but rather the high degree of heterogeneity in EV source, manufacturing approaches, dosing strategies, and reporting practices. Improved consistency and transparency in these areas, aligned with existing guidance for biological medicinal products and informed by community standards such as MISEV2023, would substantially strengthen the interpretability of clinical outcomes and facilitate regulatory evaluation.

This heterogeneity becomes particularly critical when addressing potency definition and dose justification, which represent central regulatory expectations for clinical development and market authorization of EV‐based therapeutics.

### 5.3 Potency Assays and Dosing Strategies

The development of validated potency assays is a crucial requirement for regulatory approval of EV‐based therapeutics. Unlike conventional biologics, the biological activity of EVs can be complex and non‐linear, requiring the use of context‐specific functional assays. This complexity arises from multiple factors, including the cellular origin of the vesicles, the methods used for their isolation, and ‐last but not least‐ their storage and handling conditions, all of which can significantly influence EVs content, integrity, and functionality (Susa et al. 2023). As a result, defining and standardizing potency remains one of the most critical challenges in the clinical translation of EV‐based products since the lack of standardized and validated potency assays across different laboratories remains a significant bottleneck for pharmaceutical development (Gimona et al. 2021). From a regulatory perspective, potency is not an abstract biological concept but a CQA directly linked to product identity, consistency, and clinical performance. Regulatory authorities expect potency assays to reflect the intended MoA and to be sufficiently sensitive to detect meaningful changes resulting from manufacturing variability or process modifications. This expectation is consistently reflected across international regulatory frameworks for biological medicinal products, including guidance issued by the EMA, the FDA, and the PMDA of Japan. Consistent with this expectation, both Terai et al. and the ISEV/ISCT position paper explicitly emphasize that potency testing for clinical EV products should be mechanism‐informed and aligned with a defined set of CQAs, so that potency assays remain sensitive to manufacturing variability and comparability questions across development and scale‐up (Terai et al. 2025; Giebel and Lim 2025). The MISEV2023 guidelines reaffirm the core principles established in MISEV2018 regarding the functional characterization of EVs, while emphasizing the importance of methodological rigor and contextual specificity in potency testing (Théry et al. 2018). Given the intrinsic diversity of EVs functions across *in vitro* and *in vivo* models, the guidelines do not prescribe a universal testing scheme but instead propose several general recommendations. First, physiologically relevant dose–response and time‐course studies are encouraged to better understand the specificity and kinetics of EV effects. Second, the inclusion of well‐defined negative controls is critical to distinguish between the biological activity of EVs of interest and background effects from culture media or unrelated EV populations. For instance, EVs‐depleted or unconditioned media processed identically to EVs‐containing samples can serve as appropriate baselines. Likewise, EVs from alternative cell types, unmodified parental cells, or healthy donor samples should be used as comparators depending on the experimental context. Importantly, the growing body of evidence summarized in Table S1 indicates that clinical trials frequently employ heterogeneous dosing units (e.g., particle number, protein content, volume‐based dosing), often without a clear linkage to functional potency or MoA. This lack of harmonization complicates dose justification, cross‐study comparability, and regulatory assessment, particularly in late‐stage clinical development. From a regulatory standpoint, the absence of a clear potency–dose relationship represents a critical gap that must be addressed through scientifically justified, product‐specific strategies rather than through generic or descriptive metrics alone. Recent evidence suggests that loosely associated surface components (the “corona”) and co‐isolated biomolecules may contribute to observed effects, either independently or in synergy with EVs, underscoring the need for control strategies that can disentangle these contributions. While not all control types are expected in every experimental setup, the use or development of context‐specific potency assays is strongly encouraged, particularly in preclinical and clinical applications, to ensure that observed biological activities are robust, reproducible, and attributable to EVs themselves. Additionally, determining optimal dosing and administration routes is crucial to avoid adverse effects and improve cost‐effectiveness. Dosing regimens for therapeutic EVs can be established through various *in vivo* models by evaluating circulation time, clearance rates, and therapeutic effects to identify minimal effective and optimal doses. In line with international non‐clinical guidance, dose selection for first‐in‐human studies should be supported by integrated pharmacodynamic, biodistribution, and toxicity data, with appropriate consideration of dose escalation and recovery phases where relevant.

According to FDA guidelines on potency testing for cellular and gene therapy products, both biological and non‐biological assays are recommended to assess product safety and efficacy (Food and Drug Administration 2011). However, when applied to the field of EVs, there remain substantial gaps in the reporting and formal development of such assays. To date, very few clinical trials involving EVs have incorporated validated *in vitro* or *in vivo* potency assays into their study protocols (European Medicines Agency 2023).

This limitation represents a significant regulatory challenge, as potency assays are central not only to initial clinical authorization but also to lifecycle management, including comparability assessments following manufacturing changes. Overall, the current state of potency testing and dosing strategies in EV‐based clinical development underscores the need for early, mechanism‐informed definition of CQAs and potency assays, aligned with existing international regulatory expectations for complex biologics. Addressing these aspects proactively, rather than retrospectively, will be essential to support regulatory confidence, enable meaningful dose justification, and facilitate the transition of EV‐based therapeutics from exploratory clinical studies to late‐stage development and eventual market authorization. In this context, engineered EV‐based products further emphasize the importance of mechanism‐informed potency assays and robust comparability strategies, as modifications introduced at the cellular or vesicular level may directly affect CQAs and clinical performance. These considerations can be addressed within existing regulatory expectations for complex biologics, provided that engineering‐related changes are adequately characterized and justified.

## 6 Ethical and Legal Implications

The clinical application of cell‐derived EVs raises a broad spectrum of ethical and legal considerations that must be carefully addressed within both regulatory strategies and product development plans. These considerations encompass not only the sourcing, consent, and traceability of human biological materials, but also extend to data protection, equitable access, commercialization practices, and the risks associated with unregulated or premature clinical use. As EV‐based products advance toward clinical and commercial implementation, aligning ethical oversight with evolving international legal standards is becoming increasingly important. Notably, these challenges are not unique to EVs but are shared with other complex biologically derived medicinal products, for which established ethical and legal frameworks already exist across jurisdictions. A globally coherent approach, anchored in transparency, patient safety, and scientific integrity, is essential to uphold public trust, ensure legal compliance across jurisdictions, and support ethically responsible innovation in this emerging field. In the European Union, these ethical and safety governance requirements for interventional clinical trials with EV‐based medicinal products should be read within the framework of the Clinical Trials Regulation, Regulation (EU) No 536/2014 (European Union 2014), which sets core obligations for ethics committee review, protection of subjects, safety reporting and transparency.

### 6.1 Donor Consent and Biological Material Traceability

The development of products derived from human biological materials requires stringent ethical safeguards, particularly with regard to informed donor consent and the traceability of donated substances. Directive 2004/23/EC (European Union 2004) and the EMA‐CAT 22473 guideline (European Medicines Agency 2025) establish key principles in this regard, including the obligation to ensure that donors are adequately informed about the intended therapeutic use of their biological material, its potential commercial applications, and the possibility of future use for research or product development. Where EV‐based products enter interventional clinical trials in the Union, the Clinical Trials Regulation, Regulation (EU) No 536/2014 (European Union 2014), provides the overarching legal framework for ethical authorization and safety oversight, complementing EMA guidance and relevant national rules. Donors must also be made aware of scenarios involving long‐term storage and international transfer of their material. In the European Union, these principles have been substantially reinforced and consolidated by Regulation (EU) 2024/1938 on standards of quality and safety for substances of human origin intended for human application (SoHO Regulation), which repeals Directives 2002/98/EC and 2004/23/EC and establishes a comprehensive, directly applicable legal framework covering all substances of human origin, irrespective of whether they contain living cells or are used in the manufacture of medicinal products or investigational medicinal products in clinical trials (European Union 2024).

Although these regulatory frameworks were originally developed for tissues, cells, and ATMPs, their principles are highly relevant to the emerging field of EV‐based therapies. Rather than requiring EV‐specific ethical legislation, the application of these existing consent and traceability requirements provides a robust and legally sound basis for EV‐based product development, ensuring continuity with established standards applied to other biologically derived products.

To this end, robust traceability systems should be considered essential for any clinical application of EVs. These systems must enable clear linkage from the donor to the final product and should support retrospective investigation in the case of adverse reactions or quality deviations during manufacturing. This includes the implementation of unique donor identifiers, secure electronic records, and full documentation in accordance with GMP and Good Distribution Practice (GDP) standards throughout the entire production and supply chain. Comparable traceability expectations are also embedded in non‐EU regulatory systems governing human‐derived biological materials, reinforcing the global relevance of these principles.

### 6.2 Data Protection and GDPR Compliance

The GDPR, Regulation EU 2016/679 establishes stringent requirements for the collection, processing, and sharing of personal health data across clinical and manufacturing contexts (European Union 2016). While not explicitly developed with EV‐based products in mind, the GDPR's principles are highly relevant, particularly for autologous EV applications, where donor‐specific information becomes closely integrated with processes such as biobanking, consent tracking, batch documentation, quality audits, and pharmacovigilance.

In this context, sponsors are required to uphold data minimization principles, implement secure data management systems, and maintain traceable mechanisms for managing informed consent. These obligations become especially complex in multicenter or cross‐border clinical trials, where personal data may be transferred between institutions operating under different legal frameworks. Beyond the European context, analogous data protection regimes, such as the Health Insurance Portability and Accountability Act (HIPAA) in the United States, the Act on the Protection of Personal Information (APPI) in Japan, and the Privacy Act 1988 in Australia, impose comparable obligations for the handling of sensitive health data in clinical research and therapeutic development (Conduah et al. 2025; Food and Drug Administration 2025).

Although specific guidelines for EVs are still lacking, extending GDPR‐compliant practices to EV‐based clinical development can help ensure robust protection of donor and patient privacy. This includes defining clear roles and responsibilities for data access, identifying the legal basis for processing sensitive information, and guaranteeing transparency and accountability throughout the product's lifecycle, from donation to post‐market surveillance.

### 6.3 Ethical Issues in Unregulated Use

A growing concern in the EVs field is the unauthorized clinical application of EVs, particularly in sectors such as aesthetic medicine, anti‐aging therapies, and alternative medicine. These interventions are often marketed under misleading labels such as “natural”, “minimally manipulated”, or “non‐invasive”, enabling them to bypass formal clinical trial designs, regulatory scrutiny, and ethical review mechanisms.

This unregulated landscape poses significant risks, including the creation of therapeutic misconceptions that exploit vulnerable patient populations, the absence of pharmacovigilance systems or standardized AE reporting mechanisms, and the use of unqualified or inadequately tested donor material without adherence to ethical sourcing standards.

The European Commission's 2025 report on unregulated ATMPs explicitly highlights the dangers posed by the unauthorized use of innovative biological therapies outside regulated frameworks (International Council for Harmonisation 2005). Although the report focuses primarily on ATMPs such as gene and cell therapies, many of the concerns it raises ‐particularly those related to patient safety, lack of oversight, and the erosion of public trust‐ are equally relevant to the emerging field of EVs. Comparable concerns have been raised by regulatory authorities outside the European Union, particularly by the US FDA. The FDA issued a Public Safety Notification on Exosome Products (2019) and a Consumer Alert on Regenerative Medicine Products Including Stem Cells and Exosomes (2020; content current 2024), warning about serious AEs, misleading promotional claims, and the absence of FDA‐approved exosome products for clinical use (Food and Drug Administration 2019, 2020, 2025). In addition, recent enforcement actions have continued to target the online marketing of unapproved exosome‐based products. These parallels reinforce the global need for regulatory vigilance, transparent oversight, and robust ethical governance in the clinical translation of EV‐based interventions.

### 6.4 Intellectual Property and Benefit Sharing

The development of EV‐based therapeutics frequently relies on donor‐derived cells or tissues; yet, the commercial benefits generated by these therapies seldom extend to the original donors or to the biobanks that provided the biological materials. This raises important concerns regarding distributive justice and the equitable sharing of benefits arising from biomedical innovation.

Although current EU legislation does not mandate benefit‐sharing arrangements, emerging ethical frameworks increasingly advocate for practices that enhance transparency and fairness. These frameworks encourage the explicit disclosure of potential commercial uses during the informed consent process, the exploration of voluntary benefit‐sharing models or royalty‐based agreements with public biobanks, and the involvement of stakeholders ‐such as donors and community representatives‐ through ethics committees and institutional governance boards. Best practices in this area align with the recommendations issued by international organizations like the World Health Organization (WHO) and the United Nations Educational, Scientific and Cultural Organization (UNESCO) concerning the ethical use of human biological materials, particularly in the context of first‐in‐human studies or high‐value commercial applications.

By integrating rigorous attention to ethical sourcing, robust data protection, clear regulatory oversight, and fair commercialization practices, the field of EV‐based therapeutics can build a foundation of public trust and social responsibility. As these innovative therapies move closer to clinical adoption, ensuring that development practices are ethically sound and legally compliant will be essential for the sustainable advancement of the EVs sector.

## 7 Future Directions and Recommendations

The development of cell‐derived EVs as pharmaceutical products holds transformative potential across regenerative medicine, immunotherapy, drug delivery, and diagnostics. However, realizing this promise is currently constrained by the complexity of consistently applying existing regulatory, technical, and ethical frameworks to a highly heterogeneous class of acellular, biologically derived products, rather than by the absence of regulatory instruments. Differences in interpretation across jurisdictions, together with technical variability and evolving ethical considerations, contribute to residual uncertainty in development pathways. These actions should primarily focus on the transparent, science‐driven application of existing regulatory tools for biologics and, where applicable, advanced therapies, while explicitly identifying and addressing operational grey zones that emerge in practice. A science‐driven, risk‐adapted, and patient‐centered approach must guide these developments to ensure that innovation is both effective and socially accountable. In this context, strengthening international convergence through established harmonization mechanisms and regulatory dialogue will be essential to support sustainable and responsible clinical translation of EV‐based products.

### 7.1 Establishing a Dedicated Regulatory Identity for EVs

As extensively discussed throughout this text, one of the most critical bottlenecks in the development of EV‐based products is the inconsistent interpretation and implementation of existing medicinal product frameworks across jurisdictions when applied to acellular vesicle‐based preparations. EVs may fall outside certain existing legal definitions (e.g., not substantially modified EVs not qualifying as ATMPs under current EU interpretation), yet they are developed within established pathways for biologics and related products. Accordingly, priority should be given to a transparent, case‐by‐case application of available regulatory tools (including scientific advice procedures, classification discussions, and established quality/safety paradigms), while explicitly documenting the recurring grey zones that are not yet operationally covered (e.g., criteria used for classification decisions, definition of EV‐specific CQAs, and comparability expectations).

In practical terms, these operational grey zones may arise at multiple decision points during development. Examples include determining whether a given EV‐based product should be framed primarily within biologics pathways or, where relevant, in relation to advanced‐therapy concepts; deciding how the degree of manipulation or engineering alters regulatory expectations; defining which attributes should be treated as product‐relevant CQAs in heterogeneous EV preparations; selecting potency strategies when the MoA is only partially resolved; and determining the scope of comparability data required after scale‐up, cell‐bank renewal, media changes, or bioreactor transition. In such situations, ambiguity should not be managed through ad hoc assumptions, but through structured, prospectively documented decision‐making supported by early scientific advice, explicit justification of the product's regulatory positioning, and transparent linkage between source material, manufacturing process, intended use, and the resulting quality, non‐clinical, and clinical package.

To address these practical uncertainties, a structured approach is needed to clarify how EV‐based products are positioned within existing categories and which decision criteria are applied in practice. This can be achieved by developing regulator‐facing and developer‐facing interpretative tools (e.g., “points‐to‐consider” documents and decision trees) that remain consistent with existing legislation and guidance, while specifying how key variables, such as source (autologous/allogeneic/xenogeneic), degree of manipulation, intended use, and MoA, translate into concrete quality, non‐clinical, and clinical expectations. Such clarification, supported by early and iterative regulatory dialogue (e.g., EMA Innovation Task Force interactions and national scientific advice platforms), would improve predictability for developers and promote a consistent approach across Member States without implying the necessity of new overarching regulatory frameworks (European Union 2007). In this context, the concept of a “regulatory identity” for EV‐based products should be understood as an interpretative and operational positioning within existing regulatory categories, rather than as the establishment of a new legal classification or EV‐specific regulatory framework.

Maintaining this distinction is essential to ensure alignment with current regulatory practice across jurisdictions and to avoid normative interpretations that exceed the scope of this review.

### 7.2 Harmonizing Quality Standards and Manufacturing Platforms

Quality control for EVs remains hindered by technical heterogeneity and the absence of standardized methods for assessing identity, purity, and potency. These challenges reflect the intrinsic biological complexity of EVs rather than a lack of applicable quality frameworks, and require careful adaptation of existing quality principles used for complex biologics and, where relevant, advanced therapies.

Although the MISEV2023 guidelines provide a strong scientific basis for EVs characterization, they are intended as community‐driven best practices and are not designed to function as regulatory standards.

Promoting a transparent and structured alignment between MISEV2023 principles and established regulatory quality expectations (e.g., identity, purity, potency, and process control), without implying their formal integration into regulatory frameworks, could nevertheless support greater consistency in data generation and interpretation across the field, while preserving a clear distinction between scientific guidance and legally binding requirements. Within this context, the definition of validated analytical tools, standardized reference materials, and product‐specific CQAs should be pursued using a risk‐based, fit‐for‐purpose approach consistent with existing GMP expectations.

Such efforts can be embedded within current pharmaceutical development paradigms, rather than requiring EV‐specific GMP standards. Furthermore, the development of scalable manufacturing protocols that balance clinical‐grade production with reproducibility and cost‐efficiency is crucial. Such initiatives should be actively supported by EU‐funded research programs, such as Horizon Europe, and by public‐private partnerships designed to foster sustainable innovation. At the same time, comparable support mechanisms and translational infrastructures in other regulatory regions may play a similar role in facilitating harmonized development pathways. These actions are essential to reduce technical variability, enhance regulatory confidence, and ensure that EV‐based products are developed and deployed under reliable and reproducible conditions.

### 7.3 Oversight of Compassionate use, Hospital Exemptions, Ethical Innovation and Sustainable Development Models

The non‐standard use of EVs outside clinical trials ‐through hospital exemptions, compassionate access programs, or commercial “wellness” applications raises significant regulatory and ethical concerns. Without clear guidance, these pathways risk exposing patients to unproven therapies and undermine public trust in legitimate clinical development.

To mitigate these risks, oversight of expanded access pathways should be strengthened through the consistent application of existing regulatory and ethical frameworks, rather than through the creation of EV‐specific derogations. In the European context, this includes adherence to EMA and European Commission positions on unauthorized ATMPs, which provide relevant reference principles for biologically derived products offered outside formal clinical trial settings.

Greater transparency and robust clinical justification should be required for hospital exemptions, while ethics committee review processes should ensure that decisions are grounded in established legal and ethical standards applicable to human‐derived medicinal products.

Close coordination between national competent authorities and the EMA is essential to promote a coherent and proportionate implementation of these mechanisms, ensuring that patient access is balanced with safety, scientific validity, and regulatory accountability. This alignment would reinforce the credibility of EV‐based therapies and promote ethical, patient‐centered clinical development.

The sourcing and commercialization of EV‐based therapies must adhere to ethical principles grounded in transparency, informed consent, and equitable benefit‐sharing. Particular attention is required when biological material is sourced from public biobanks or from donors who may not directly benefit from the clinical applications developed from their donations. To uphold these principles, informed consent and governance frameworks should be implemented in line with existing European regulations, including Directive 2004/23/EC (European Union 2004) and the GDPR, without introducing EV‐specific ethical requirements that would diverge from established practice. Encouraging voluntary benefit‐sharing schemes and adopting ethical commercialization practices can further ensure that innovation serves broader societal interests. Additionally, actively involving ethics committees and patient representatives in decision‐making processes, particularly for first‐in‐human trials and pediatric applications, is crucial to safeguarding donor rights and maintaining public trust. Building such transparent and inclusive governance models will be pivotal in securing the long‐term sustainability and legitimacy of EV‐based pharmaceutical development.

### 7.4 Advancing International Regulatory Harmonization

Given the globalized nature of pharmaceutical development, international coordination is vital to avoid duplicative efforts, streamline product development, and facilitate multinational clinical trials. While agencies such as the EMA, FDA, and PMDA have begun addressing EV‐based products within broader frameworks applicable to biologics and, where relevant, advanced therapies, differences in interpretation and implementation persist across jurisdictions. In this context, international harmonization efforts should prioritize the consistent application of existing ICH guidelines as a shared baseline for quality, non‐clinical, and clinical development, rather than the creation of EV‐specific regulatory instruments. Where EV‐related scientific or operational questions are not yet fully addressed within existing guidance, aligned “points‐to‐consider” documents or Q&A formats, developed through established harmonization platforms, potentially including ICH or WHO convening mechanisms, could support convergence while remaining fully consistent with current regulatory frameworks. Aligning quality, safety, and clinical trial standards for EVs across major regulatory agencies‐including the EMA, FDA, PMDA, Health Canada, MHRA, and TGA‐would facilitate a consistent global regulatory landscape. Collaboration with scientific consortia such as the ISEV, the International Society for Cell and Gene Therapy (ISCT‐ https://www.asgct.org), and the Alliance for Regenerative Medicine (ARM‐ https://alliancerm.org) would further strengthen these efforts. Such convergence would not imply the establishment of a unified or EV‐specific global regulatory framework, but rather a progressive refinement and alignment of existing regulatory practices across regions. In this way, regulatory foresight, ethical responsibility, and scientific excellence can collectively guide the future development of EV‐based pharmaceuticals within established systems, ensuring that therapeutic innovation remains effective, proportionate, and socially responsible. A summary of the major challenges and corresponding strategic recommendations is provided in Table 2.

**TABLE 2:** Key regulatory and developmental challenges for EV‐based therapeutics and proposed strategic actions.

Challenge area	Specific issue	Recommended action
Regulatory classification	Uncertainty in the classification of EV‐based products within existing regulatory categories, particularly for acellular and non‐substantially modified products	Clarify and consistently apply existing regulatory frameworks (e.g., biologics, ATMPs where applicable) through early scientific advice and regulatory dialogue with competent authorities
Quality and characterization	Challenges in defining product‐specific identity, purity, and potency attributes due to EV heterogeneity	Apply existing quality frameworks for complex biologics using fit‐for‐purpose analytical strategies, supported by community‐driven best practices for EV characterization
GMP compliance and scalability	Adaptation of established GMP principles to EV manufacturing, including scalability and process control challenges	Implement GMP‐compliant manufacturing workflows based on existing guidance for biologics and ATMPs, with process validation, closed systems, and full traceability
Safety and risk management	Potential safety risks related to immunogenicity, off‐target effects, and biodistribution profiles	Apply risk‐based safety assessment strategies used for advanced therapies and complex biologics, including quality‐linked CQAs, biodistribution studies, and toxicological evaluation
Clinical development	Variability in clinical study design, dosing rationale, and potency assessment across EV‐based trials	Develop context‐specific potency assays and dosing strategies aligned with intended use, supported by harmonized clinical endpoints and transparent reporting
Ethical/legal oversight	Ethical and legal challenges related to donor consent, traceability, data protection, and benefit sharing	Ensure compliance with existing ethical and legal frameworks for human‐derived products, including informed consent, GDPR‐compliant data handling, and transparent commercialization practices
Global harmonization	Fragmented interpretation and implementation of existing guidance across regulatory agencies, leading to variability in expectations for quality, non‐clinical packages, and clinical documentation	Promote convergence through existing international harmonization mechanisms (e.g., ICH guidelines and established WHO frameworks), supported by transparent cross‐agency dialogue and consistent scientific advice pathways

## 8 Conclusion

Cell‐derived EVs represent one of the most promising innovations in contemporary pharmaceutical research. Their intrinsic biocompatibility, molecular versatility, and capacity to mediate intercellular communication position them as highly attractive candidates for a wide range of therapeutic and diagnostic applications, including immunomodulation, regenerative medicine, targeted drug delivery, and biomarker discovery.

Despite this broad potential, the path to clinical translation remains challenging, primarily due to the complexity of consistently interpreting and applying existing regulatory frameworks to EV‐based products, combined with limited EV‐focused operational clarification in areas such as classification decision criteria, critical quality attribute definition, potency assessment, and comparability. Currently, EVs are situated at the interface between existing categories such as biologics and ATMPs. According to the EC regulation No 1394/2007, ATMPs comprise gene therapy medicinal products, somatic cell therapy medicinal products, tissue‐engineered products, and combined ATMPs. These categories were designed to address the complexity of therapies involving living cells and tissues. However, the most recent EMA guideline on investigational ATMPs explicitly clarifies that “not substantially modified EVs and cellular fragments originating from human cells or chemically synthesized therapeutic sequences do not fulfil the current definition of ATMPs and therefore the risk‐based approach as laid out in Regulation (EC) No 1394/2007 does not apply.” For further detail, reference is made to the Reflection Paper on ATMP classification (European Medicines Agency 2015). This regulatory positioning does not imply the absence of applicable regulatory pathways for EV‐based products, but rather highlights the need for transparent and consistent application of existing frameworks to acellular, biologically active entities that share scientific and manufacturing challenges with cell‐ and gene‐based therapies. These include heterogeneity, complex mechanisms of action, and the need for validated, scalable production methods and potency assays. Variability in interpretation across jurisdictions may contribute to divergent regulatory expectations and development strategies, potentially increasing uncertainty for both developers and regulators.

At the same time, the scientific community has made significant progress in systematizing EVs research through shared standards and expert consensus initiatives, such as the MISEV2023. These efforts, coordinated under the auspices of the ISEV, offer a solid foundation for regulatory discussion.

While such community‐driven frameworks are not intended to function as regulatory standards, they provide an important scientific reference that can support regulatory evaluation when appropriately contextualized.

This review underscores the importance of fostering closer alignment between scientific advances and the regulatory interpretation of existing quality, safety, and efficacy requirements. The development of a clearer and consistently applied regulatory positioning for EV‐based products, through transparent interpretative guidance and early, iterative regulatory dialogue, could serve as a critical enabler for safe, reproducible, and ethically grounded clinical translation. Such integration would require the coordinated engagement of multiple stakeholders: regulatory authorities, academic researchers, industry developers, ethics committees, and international organizations.

Efforts to address current uncertainties should be grounded in existing regulatory instruments and scientific knowledge, while explicitly acknowledging and documenting operational grey zones where further clarification may be beneficial. Establishing a clearer regulatory identity for EVs may contribute to reducing development barriers and could facilitate public health objectives by improving access to innovative, patient‐centered therapies developed in accordance with the highest scientific and ethical standards. The considerations presented herein are not intended as normative or prescriptive recommendations, but rather as a constructive contribution to an ongoing international dialogue on the clinical development and regulation of EV‐based therapeutics. This perspective is offered in the hope of supporting and encouraging further discussion among regulatory authorities, scientific organizations, and industrial stakeholders, with the shared objective of enabling the responsible and effective translation of EV‐based products from research to clinical practice.

## Author Contributions

**Tania Limongi**: conceptualization, writing – original draft, writing – review and editing, data curation, supervision, resources. **Paola Milla**: writing – original draft, writing – review and editing, data curation. **Barbara Stella**: writing – review and editing, writing – original draft. **Silvia Arpicco**: writing – original draft, writing – review and editing, resources.

## Conflicts of Interest

The author Tania Limongi is co‐inventors on patent WO 2023/073609 Title: Method for obtaining concentrated population of extracellular vesicles washed of the physiopathological load thereof.

## Supporting information




**Supporting Information**: jev270332‐sup‐0001‐TableS1.docx

## Data Availability

The authors have nothing to report.
