# Supplementary material for: Regulatory Challenges and Opportunities for Cell‐Derived Extracellular Vesicles in Pharmaceutical Development: A European and Global Perspective
Source: J Extracell Vesicles. 2026 Jun 29;15(7):e70332. doi: 10.1002/jev2.70332 (PMC13314385; doi:10.1002/jev2.70332)
Supplement: Supplementary file 1 — Supporting Information: jev270332‐sup‐0001‐TableS1.docx [file JEV2-15-e70332-s001.docx]

Supplementary Table S1. Full list of active and completed clinical trials investigating the therapeutic potential of extracellular vesicles (EVs) across multiple disease areas. Trials include a variety of EV sources (e.g., mesenchymal stem cells, blood derivatives, neural or adipose tissues) and cover a wide range of conditions from oncology to neurodegeneration, inflammatory, metabolic, and respiratory disorders.

The list was obtained through a search on the ClinicalTrials.gov website (last access: 4 February 2026) using the following queries: “exosomes” OR “extracellular vesicles”, and filtering for “interventional” studies only. The 252 studies identified by the search were then manually screened to exclude those in which exosomes were used for diagnostic or cosmetic purposes, as well as those that had been prematurely withdrawn.

Link to the search: https://clinicaltrials.gov/search?cond=%22exosomes%22%20OR%20%22extracellular%20vesicles%22&aggFilters=studyType:int

Abbreviations: ARDS = Acute Respiratory Distress Syndrome; ALS = Amyotrophic Lateral Sclerosis; BPD = Bronchopulmonary Dysplasia; EV = Extracellular Vesicle; HUC = Human Umbilical Cord; LSC = Limbal Stem Cell; MSC = Mesenchymal Stem Cell; N.A. = Not Applicable or Not Available; POF = Premature Ovarian Failure; KOA = Knee Osteoarthritis; sEV = small Extracellular Vesicle; PEP = Platelet-derived Extracellular Product; PRP = Platelet-rich Plasma; PSC = Pluripotent Stem Cell; UC = Umbilical Cord .

| **Disease category** | **Study Title** | **Phase** | **Study Status** | **Source of EVs** | **Sponsor** | **NCT Number** |
| --- | --- | --- | --- | --- | --- | --- |
| **Cancer** | Clinical Study on the Safety and Efficacy of BiTE-EV in Relapsed/Refractory Acute B-Cell Leukemia | I | Recruiting | Mesenchymal stem cells | Beijing GoBroad Hospital | NCT06890494 |
|  | Safety and Efficacy of ZZSW-01 in Relapsed/Refractory B-cell Malignancies | I | Recruiting | extracellular vesicle vector that carries CD19 CAR mRNA | Union Hospital, Tongji Medical College, Huazhong University of Science and Technology | NCT07240974 |
|  | UCMSC-Exo for Chemotherapy-induced Myelosuppression in Acute Myeloid Leukemia | I | Recruiting | Umbilical cord mesenchymal stem cells | Union Hospital, Tongji Medical College, Huazhong University of Science and Technology | NCT06245746 |
|  | iExosomes in Treating Participants With Metastatic Pancreas Cancer With KrasG12D Mutation | I/II | Recruiting | Mesenchymal Stromal Cells-derived Exosomes with KRAS G12D siRNA | M.D. Anderson Cancer Center | NCT03608631 |
| **Cardiovascular diseases** | Treatment of Non-ischemic Cardiomyopathies by Intravenous Extracellular Vesicles of Cardiovascular Progenitor Cells | I | Recruiting | Cardiovascular progenitor cells | Assistance Publique - Hôpitaux de Paris | NCT05774509 |
|  | Safety Evaluation of Intracoronary Infusion of Extracellular Vesicles in Patients Following Coronary Stent Implantation | I | Completed | Platelet enriched blood | Christopher J. McLeod | NCT04327635 |
| **Digestive system diseases** | MSC-EVs in Acute/ Acute-on-Chronic Liver Failure After Liver Transplantation | I | Not yet recruiting | Mesenchymal stem cells | Third Affiliated Hospital, Sun Yat-Sen University | NCT06825572 |
|  | Effect of Mesenchymal Stem Cells-derived Exosomes in Decompensated Liver Cirrhosis | II | Unknown | Mesenchymal stem cells | Research Institute for Gastroenterology and Liver Diseases (RIGLD) | NCT05871463 |
|  | Using Autologous Bone Marrow Cells With Wharton Gel Exosomes to Stimulate Hepatic Cell Repair in Liver Cirrhosis (bone marrow) | N.A. | Not yet recruiting | Wharton's jelly mesenchymal stem cells | Global Stem Cell Center, Baghdad | NCT07374185 |
|  | National Collaborative Centre for Hepatic Regenerative Medicine (NC-CHRM): Evaluating Mesenchymal Stem Cell Therapy in Non-viral Acute on Chronic Liver Failure (ACLF) Patient- Phase-II Trial | II | Not yet recruiting | Umbilical cord mesenchymal stem cells | Institute of Liver and Biliary Sciences, India | NCT07131306 |
|  | DB-3Q bmMSC-EVs in Patients With Perianal Fistulizing Crohn's Disease | II | Recruiting | Bone marrow mesenchymal stem cell | Direct Biologics, LLC | NCT06918808 |
|  | Study of ExoFlo for the Treatment of Medically Refractory Crohn's Disease | I | Terminated | Bone marrow mesenchymal stem cell | Direct Biologics, LLC | NCT05130983 |
|  | Safety of Injection of Placental Mesenchymal Stem Cell Derived Exosomes for Treatment of Resistant Perianal Fistula in Crohn's Patients | I/II | Unknown | Placental mesenchymal stem cell | Tehran University of Medical Sciences | NCT05499156 |
|  | Study of ExoFlo for the Treatment of Medically Refractory Ulcerative Colitis | I | Terminated | Bone marrow mesenchymal stem cell | Direct Biologics, LLC | NCT05176366 |
|  | hucMSCs Exosomes for the Treatment of Active Ulcerative Colitis | I | Not yet recruiting | Umbilical cord mesenchymal stem cells | Shanghai East Hospital | NCT06853522 |
|  | Safety and Efficacy of Injection of Human Placenta Mesenchymal Stem Cells Derived Exosomes for Treatment of Complex Anal Fistula | I/II | Unknown | Placental mesenchymal stem cell | Tehran University of Medical Sciences | NCT05402748 |
|  | Study of ExoFlo for the Treatment of Perianal Fistulas | I/II | Terminated | Bone marrow mesenchymal stem cell | Direct Biologics, LLC | NCT05836883 |
|  | Human Placenta Mesenchymal Stem Cells Derived Exosomes Injection for Treatment of Complex Anal Fistula | II | Not yet recruiting | Mesenchymal stem cells | Tehran University of Medical Sciences | NCT06568653 |
|  | Effects of Exosome Administration in Preventing Early Leakage in Rectal Cancer Patients Undergoing Low Anterior Resection | I | Not yet recruiting | Mesenchymal stem cells | Tehran University of Medical Sciences | NCT06536712 |
| **Gynecological diseases** | Intra-ovarian Injection of MSC-EVs in Idiopathic Premature Ovarian Failure | I/II | Recruiting | Bone marrow mesenchymal stem cell | Royan Institute | NCT06202547 |
|  | The Use of Expanded Mesenchymal Stromal Cells (MSC) in Premature Ovarian Failure (POF) in Adult Humans | I | Active not recruiting | Autologous bone marrow mesenchymal stem cell | University of Jordan | NCT04815213 |
|  | Evaluating Mechanically Engineered Stem Cell Exosomes for Treating Endometrial Injury: A Clinical Study | I/II | Recruiting | Umbilical cord mesenchymal stem cells | Tang-Du Hospital | NCT06896747 |
|  | MSC Exosome Therapy for Post-Preeclampsia Endothelial Dysfunction | I/II | Recruiting | Mesenchymal stem cells | Universitas Padjadjaran | NCT07183384 |
| **Urogenital Diseases** | Clinical Trial Investigating the Effect of Exosomes as a Complementary Treatment in Severe to Moderate Erectile Dysfunction | II | Not yet recruiting | Umbilical cord mesenchymal stem cells | Labbafinejad Medical Center | NCT07319533 |
|  | PRP Exosomes Therapy for Erectile Dysfunction | II | Recruiting | Platelet enriched blood | Institute for the Study of Urological Diseases, Greece | NCT07124871 |
| **Musculoskeletal diseases** | Treatment of Patients With Bone Tissue Defects Using Mesenchymal Stem Cells Enriched by Extracellular Vesicles | I/II | Unknown | Mesenchymal stem cells | Institute of Biophysics and Cell Engineering of National Academy of Sciences of Belarus | NCT05520125 |
|  | The Effect of Human Umbilical Cord Mesenchymal Stem Cells and Exosomes on the Healing of Postoperative Pain and Periapical Lesions in the Treatment of Apical Periodontitis: Randomized Controlled Clinical Study | I/II | Not yet recruiting | Umbilical cord mesenchymal stem cells | TC Erciyes University | NCT06764004 |
|  | Evaluation of Adipose Derived Stem Cells Exo.in Treatment of Periodontitis | I | Unknown | Adipose tissue | Beni-Suef University | NCT04270006 |
|  | Safety and Preliminary Efficacy of iEVs Injection in Treating Lateral Epicondylitis of Humerus | I/II | Recruiting | Mesenchymal stem cells | Shanghai 6th People's Hospital | NCT07111325 |
|  | Intra-articular Injection of UC-MSC Exosome in Knee Osteoarthritis | I | Recruiting | Umbilical cord mesenchymal stem cells | Universidad de los Andes, Chile | NCT06431152 |
|  | Phase 1b Clinical Trial to Evaluate PEP and EUFLEXXA for Knee Osteoarthritis (KOA) | I | Not yet recruiting | Platelet enriched blood | Rion Inc. | NCT06463132 |
|  | Use of Extracellular Vesicles (EV) for Knee Osteoarthrosis | I | Recruiting | Mesenchymal stem cells | University of Jordan | NCT06937528 |
|  | Mesenchymal Stem Cells Derived Exosomes in Osteoarthritis Patients | N.A. | Recruiting | Mesenchymal stem cells | Isfahan University of Medical Sciences | NCT06466850 |
|  | Clinical Efficacy of Exosome in Degenerative Meniscal Injury (KNEEXO) | II | Unknown | Mesenchymal stem cells | Eskisehir Osmangazi University | NCT05261360 |
|  | Safety and Feasibility of Umbilical Cord Wharton's Jelly Allograft Injections for Lumbar Pain | I | Recruiting | Wharton's jelly (umbilical) mesenchymal stem cells | R3 Stem Cell | NCT06361485 |
| **Neurological diseases** | Safety of Cultured Allogeneic Adult Umbilical Cord Stem Cells Exosomes for Trigeminal Neuralgia | I | Recruiting | Umbilical cord mesenchymal stem cells | The Foundation for Orthopaedics and Regenerative Medicine | NCT05152368 |
|  | HUC-MSC-sEV-001 Nasal Drops for Neurodegenerative Diseases | I | Not yet recruiting | Umbilical cord mesenchymal stem cells | Xuanwu Hospital, Beijing | NCT06607900 |
|  | Exploring Nasal Drop Therapy With Small Extracellular Vesicles for Amyotrophic Lateral Sclerosis (ALS) | I/II | Recruiting | Umbilical cord mesenchymal stem cells | Xuanwu Hospital, Beijing | NCT06598202 |
|  | The Safety and Efficacy of NouvSoma001 in Neuromyelitis Optica Spectrum Disorders | I | Recruiting | Neural stem cell | Tongji Hospital | NCT06620809 |
|  | The Pilot Experimental Study of the Neuroprotective Effects of Exosomes in Extremely Low Birth Weight Infants | N.A. | Not yet recruiting | Mesenchymal stem cells | Federal State Budget Institution Research Center for Obstetrics, Gynecology and Perinatology Ministry of Healthcare | NCT05490173 |
|  | Adia MED of Winter Park LLC Autism Spectrum Disorder Research Study | I/II | Recruiting | Umbilical cord mesenchymal stem cells | Adia Med of Winter Park LLC | NCT07304440 |
|  | Prospective Clinical Study on Human Umbilical Cord Mesenchymal Stem Cell-Derived Exosomes for the Treatment of Childhood Autism | I/II | Recruiting | Umbilical cord mesenchymal stem cells | Dongfang People's Hospital | NCT07243561 |
|  | Combined Photo-Biomodulation At Acupuncture Points, Autologous PRP, and Umbilical Cord-Derived Exosome Therapy in Autism Spectrum Disorder (Biomodulation) | N.A. | Recruiting | Umbilical cord mesenchymal stem cells | Iffat Anwar Medical Complex | NCT06600529 |
|  | Induced Pluripotent Stem Cell Derived Exosomes Nasal Drops for the Treatment of Refractory Focal Epilepsy | I | Unknown | Induced pluripotent stem cell | Peking Union Medical College Hospital | NCT05886205 |
|  | Extracellular Vesicles for the Treatment of Syringomyelia | I | Recruiting | Mesenchymal stem cells | Xuanwu Hospital, Beijing | NCT07295067 |
|  | Allogeneic Mesenchymal Stem Cell-Derived Exosome Therapy for Progressive Multiple Sclerosis | I | Suspended | Mesenchymal stem cells | Biocells Medical | NCT07146087 |
|  | Patients With ALS and Other Motor Disorders Will be Treated With Mesenchymal Cell Exosome Solution | I | Completed | Mesenchymal stem cells | The Foundation for Orthopaedics and Regenerative Medicine | NCT07105371 |
|  | Safety of Cultured Allogeneic Adult Umbilical Cord Derived Mesenchymal Stem Cell Exosomes for Parkinson's Disease | I | Recruiting | Mesenchymal stem cells | The Foundation for Orthopaedics and Regenerative Medicine | NCT05152394 |
|  | Induced Pluripotent Stem Cells-derived Small Extracellular Vesicles May Promote Neurological Function and Improve Cognitive Impairment in Acute Ischemic Stroke Patients | I/II | Recruiting | Mesenchymal stem cells | Hongmei Wang | NCT07145294 |
|  | The Safety and Efficacy of NouvSoma001 in Ischemic Stroke | I | Recruiting | Neural stem cell | Tongji Hospital | NCT06612710 |
|  | Exploratory Study of Intranasal Administration of Human Umbilical Cord Mesenchymal Stem Cell-Derived Extracellular Vesicles for the Treatment of Ischemic Stroke | I | Not yet recruiting | Umbilical cord mesenchymal stem cells | Zhujiang Hospital | NCT07232563 |
|  | Human Induced Neural Stem Cell-derived Exosomes for Treating Acute Ischemic Stroke (NSAIS) | I/II | Not yet recruiting | Neural stem cell | Xuanwu Hospital, Beijing | NCT07143786 |
|  | The STem Cell-derived Extracellular Vesicle Therapy In Acute Ischemic Stroke (STEVIA) | II | Not yet recruiting | Mesenchymal stem cells | S&Ebio Co. Ltd. | NCT06995625 |
|  | The Effect of GD-iExo-003 in Acute Ischemic Stroke (ExoCURE) | I/II | Recruiting | Human induced pluripotent stem cell | Xuanwu Hospital, Beijing | NCT06138210 |
|  | hUC-MSC-Exo Therapy for Autoimmune Encephalitis | I/II | Not yet recruiting | Umbilical cord mesenchymal stem cells | Xuanwu Hospital, Beijing | NCT07131683 |
|  | Patients With Congenital Myasthenic Syndrome Will be Treated With Mesenchymal Stem Cell Exosome Solution | I | Recruiting | Mesenchymal stem cells | The Foundation for Orthopaedics and Regenerative Medicine | NCT07226726 |
| **Nutritional & metabolic diseases** | Preliminary Safety and Efficacy Study of Extracellular Vesicle Infusion in the Intervention of Age-related Phenotypes With Impaired Glucose Tolerance. | N.A. | Not yet recruiting | Wharton's jelly mesenchymal stem cells | BEAUTECH | NCT06495437 |
|  | Mesenchymal Stromal/Stem Cells-Derived Exosomes (MSC-Exos) Therapy for Type 2 Diabetes Mellitus Patients | II | Not yet recruiting | Mesenchymal stem cells | Indonesia University | NCT07144241 |
| **Ophthalmic diseases** | Safety and Efficacy of Stem Cell Small Extracellular Vesicles in Patients With Retinitis Pigmentosa | I/II | Recruiting | Mesenchymal stem cells | Mahidol University | NCT06242379 |
|  | The Effect of Stem Cells and Stem Cell Exosomes on Visual Functions in Patients With Retinitis Pigmentosa | II/III | Unknown | Mesenchymal stem cells | TC Erciyes University | NCT05413148 |
|  | Exosomes in Patients With Ocular Involvement of Sjögren's Syndrome | I | Not yet recruiting | Mesenchymal stem cells | Guangdong ProCapZoom Biosciences Co., Ltd. | NCT07192666 |
|  | Limbal Stem Cell Derived Exosome (LSC-Exo) Eye Drop for Treatment of Dry Eye (EXOIUMS) | I | Recruiting | Limbal Stem Cell | Iran University of Medical Sciences | NCT06543667 |
|  | Safety and Efficacy of Pluripotent Stem Cell-derived Mesenchymal Stem Cell Exosome (PSC-MSC-Exo) Eye Drops Treatment for Dry Eye Diseases Post Refractive Surgery and Associated With Blepharospasm | I/II | Unknown | Mesenchymal stem cells | Second Affiliated Hospital, School of Medicine, Zhejiang University | NCT05738629 |
|  | Effect of UMSCs Derived Exosomes on Dry Eye in Patients With cGVHD | I/II | Unknown | Mesenchymal stem cells | Zhongshan Ophthalmic Center, Sun Yat-sen University | NCT04213248 |
|  | Effect of PAE Hydrogel Loaded With tRF-ASO-Exo on Patients With Diabetic Ocular Surface Diseases | II | Not yet recruiting | Mesenchymal stem cells | Affiliated Hospital of Nantong University | NCT07382453 |
| **Otorhinolaryngological diseases** | Autologous Blood Monocyte Vesicles for the Treatment of Sudden Deafness | I | Not yet recruiting | Autologous blood monocyte | Sun Yat-Sen Memorial Hospital of Sun Yat-Sen University | NCT06707389 |
|  | Intracochlear Application of VSF1.01 for the Reduction of Cochlear Implant Surgery Related Trauma | I/II | Recruiting | Umbilical cord mesenchymal stem cells | Hannover Medical School | NCT06545175 |
|  | Use of Autologous Plasma Rich in Platelets and Extracellular Vesicles in the Surgical Treatment of Chronic Middle Ear Infections | II/III | Unknown | Autologous platelet- and extracellular vesicle-rich plasma | University Medical Centre Ljubljana | NCT04761562 |
|  | Efficacy of Platelet- and Extracellular Vesicle-rich Plasma in Chronic Postsurgical Temporal Bone Inflammations | N.A. | Completed | Autologous platelet- and extracellular vesicle-rich plasma | University Medical Centre Ljubljana | NCT04281901 |
| **Respiratory diseases** | Extracellular Vesicles From Mesenchymal Cells in the Treatment of Acute Respiratory Failure | I/II | Not yet recruiting | Mesenchymal stem cells | D'Or Institute for Research and Education | NCT06002841 |
|  | Extracellular Vesicle Treatment for Acute Respiratory Distress Syndrome (ARDS) (EXTINGUISH ARDS) | III | Recruiting | Bone marrow mesenchymal stem cell | Direct Biologics, LLC | NCT05354141 |
|  | Extracellular Vesicle Infusion Treatment for COVID-19 Associated ARDS | II | Completed | Bone marrow mesenchymal stem cell | Direct Biologics, LLC | NCT04493242 |
|  | A Clinical Study of Mesenchymal Stem Cell Exosomes Nebulizer for the Treatment of ARDS | I/II | Completed | Mesenchymal stem cells | Ruijin Hospital | NCT04602104 |
|  | A Clinical Study on Safety and Effectiveness of Mesenchymal Stem Cell Exosomes for the Treatment of COVID-19. | I | Unknown | Mesenchymal stem cells | First Affiliated Hospital of Wenzhou Medical University | NCT05787288 |
|  | A Safety Study of IV Stem Cell-derived Extracellular Vesicles (UNEX-42) in Preterm Neonates at High Risk for Bronchopulmonary Dysplasia (BPD) | I | Terminated | Bone marrow mesenchymal stem cell | United Therapeutics | NCT03857841 |
|  | Safety and Efficacy of Umbilical Cord Mesenchymal Stem Cell Exosomes in Treating Chronic Cough After COVID-19 | I | Unknown | Umbilical cord mesenchymal stem cells | Huazhong University of Science and Technology | NCT05808400 |
|  | Safety and Efficacy of MSC-EVs in the Prevention of Bronchopulmonary Dysplasia (BPD) in Extremely Preterm Infants | I/II | Recruiting | Mesenchymal stem cells | EXO Biologics S.A. | NCT06279741 |
|  | Inhaled Mesenchymal Stem Cell-Derived Exosomes in the Treatment of Post-Infectious Cough: A Single-Center Randomized Controlled Clinical Trial | I/II | Recruiting | Mesenchymal stem cells | Shanghai Changzheng Hospital | NCT07103980 |
|  | Nebulized MSC-Exos for Anti-MDA5+ RP-ILD: Safety and Efficacy Trial | I | Recruiting | Mesenchymal stem cells | Li Shiyue | NCT06919380 |
| **Skin & connective tissue diseases** | Exosome Treatment in Androgenetic Alopecia | III | Completed | Foreskin-derived mesenchymal stem cells | Yeditepe University Hospital | NCT06539273 |
|  | A Split-Scalp Study Evaluating the Efficacy and Safety of Fractional Laser Therapy With and Without Exosomes in the Treatment of Androgenetic Alopecia | N.A. | Not yet recruiting | Umbilical cord mesenchymal stem cells | Chang Gung Memorial Hospital | NCT07248410 |
|  | Efficacy and Safety Of AGE ZERO™ EXOSOMES To Treat Men and Women With Androgenetic Alopecia | I | Not yet recruiting | Wharton's jelly (umbilical) mesenchymal stem cells | Levit Dermatology | NCT06482541 |
|  | Evaluation of Exosomes Injection in Treatment of Male Androgenetic Alopecia: Clinical and Immunohistochemical Study | I/II | Not yet recruiting | Plasma derived exosomes | Ain Shams University | NCT07112586 |
|  | Umbilical Cord Mesenchymal Stem Cell-Derived Exosomes in the Treatment of Androgenic Alopecia | N.A | Recruiting | Umbilical cord mesenchymal stem cells | Fujian Medical University Union Hospital | NCT07373054 |
|  | Exosome Effect on Prevention of Hairloss | N.A. | Unknown | Amniotic mesenchymal stem cells | Isfahan University of Medical Sciences | NCT05658094 |
|  | Exosomes for Hairloss Treatment | I | Not yet recruiting | Mesenchymal stem cells | Guangzhou Bio-gene Technology Co., Ltd | NCT06932393 |
|  | Aerosolized Mesenchymal Stem Cell-Derived Exosomes for the Prevention and Treatment of Radiation-Induced Oral Mucositis | II | Not yet recruiting | Mesenchymal stem cells | First Affiliated Hospital of Guangxi Medical University | NCT07312656 |
|  | Study to Evaluate the Safety, Tolerability, and Efficacy of PEP-TISSEEL in Subjects With Chronic Radiation Ulcer | I/II | Not yet recruiting | Platelet enriched blood | Rion Inc. | NCT06793748 |
|  | Use of Extracellular Vesicles (EV) for Diabetic Foot Ulcers | I | Active not recruiting | Mesenchymal stem cells | University of Jordan | NCT06825884 |
|  | Efficacy and Safety of Wharton's Jelly-Derived Mesenchymal Stem Cell Exosomes in the Treatment of Diabetic Foot Ulcers: A Double-blinded Randomized Controlled Clinical Trial | I | Completed | Wharton's jelly (umbilical) mesenchymal stem cells | Kafrelsheikh University | NCT06812637 |
|  | Safety of Extracellular Vesicles for Burn Wounds | I | Completed | Bone marrow mesenchymal stem cell | Aegle Therapeutics | NCT05078385 |
|  | Pilot Study of Human Adipose Tissue Derived Exosomes Promoting Wound Healing | N.A. | Completed | Adipose tissue | Shanghai Ninth People's Hospital Affiliated to Shanghai Jiao Tong University | NCT05475418 |
|  | Randomized, Controlled, Multicenter Study of Extracellular Vesicles From Human Adipose Tissue Promoting Wound Healing | N.A. | Recruiting | Adipose tissue | Shanghai Ninth People's Hospital Affiliated to Shanghai Jiao Tong University | NCT06253975 |
|  | Autologous Serum-derived EV for Venous Trophic Lesions Not Responsive to Conventional Treatments | N.A. | Unknown | Autologous extracellular vesicles from serum | University of Turin, Italy | NCT04652531 |
|  | The Role of Mesenchymal Stem Cell and Exosome in Treating Pilonidal Sinus Disease in Children | N.A. | Recruiting | Mesenchymal stem cells | Mustafa Azizoğlu | NCT06391307 |
|  | PEP on a Skin Graft Donor Site Wound | I | Unknown | Platelet enriched blood | Rion Inc. | NCT04664738 |
|  | Induced Pluripotent Stem Cell Derived Exosomes Nasal Drops for the Treatment of Stable Vitiligo | I | Not yet recruiting | Mesenchymal stem cells | Second Affiliated Hospital of Wannan Medical College | NCT06810869 |
|  | Use of Allogeneic Extracellular Secretomes (EV)Derived From Umbilical Cord Mesenchymal Stromal Cells in stable non-facial vitiligo: a Phase I Open-label Safety Trial. | I | Active not recruiting | Umbilical cord mesenchymal stem cells | University of Jordan | NCT06813027 |
|  | MSC EVs in Dystrophic Epidermolysis Bullosa | I/II | Recruiting | Bone marrow mesenchymal stem cell | Aegle Therapeutics | NCT04173650 |
|  | Effect of Ev.FV on Wound Healing in Dystrophic Epidermolysis Bullosa | I/II | Recruiting | Mesenchymal stem cells | Isfahan University of Medical Sciences | NCT07230223 |
|  | A Study to Investigate the Safety, Tolerability, and Efficacy of BxC-I17e Single and Multiple Dose SC Injection in Patients With Moderate to Severe Atopic Dermatitis | I | Recruiting | Mesenchymal stem cells | Brexogen Inc. | NCT06055361 |
|  | Safety and Tolerability Study of MSC Exosome Ointment | I | Completed | Mesenchymal stem cells | Paracrine Therapeutics Dermatology Pte. Ltd. | NCT05523011 |
